# Patterns of brain volume and metabolism predict clinical features in the progressive supranuclear palsy spectrum

**DOI:** 10.1093/braincomms/fcae233

**Published:** 2024-07-16

**Authors:** Farwa Ali, Heather Clark, Mary Machulda, Matthew L Senjem, Val J Lowe, Clifford R Jack, Keith A Josephs, Jennifer Whitwell, Hugo Botha

**Affiliations:** Department of Neurology, Mayo Clinic, Rochester, MN 55905, USA; Department of Neurology, Mayo Clinic, Rochester, MN 55905, USA; Department of Psychiatry and Psychology, Mayo Clinic, Rochester, MN 55905, USA; Department of Radiology, Mayo Clinic, Rochester, MN 55905, USA; Department of Radiology, Mayo Clinic, Rochester, MN 55905, USA; Department of Radiology, Mayo Clinic, Rochester, MN 55905, USA; Department of Neurology, Mayo Clinic, Rochester, MN 55905, USA; Department of Radiology, Mayo Clinic, Rochester, MN 55905, USA; Department of Neurology, Mayo Clinic, Rochester, MN 55905, USA

**Keywords:** neurodegeneration, tauopathy, machine learning, diagnosis, neuroimaging

## Abstract

Progressive supranuclear palsy (PSP) is a neurodegenerative tauopathy that presents with highly heterogenous clinical syndromes. We perform cross-sectional data-driven discovery of independent patterns of brain atrophy and hypometabolism across the entire PSP spectrum. We then use these patterns to predict specific clinical features and to assess their relationship to phenotypic heterogeneity. We included 111 patients with PSP (60 with Richardson syndrome and 51 with cortical and subcortical variant subtypes). Ninety-one were used as the training set and 20 as a test set. The presence and severity of granular clinical variables such as postural instability, parkinsonism, apraxia and supranuclear gaze palsy were noted. Domains of akinesia, ocular motor impairment, postural instability and cognitive dysfunction as defined by the Movement Disorders Society criteria for PSP were also recorded. Non-negative matrix factorization was used on cross-sectional MRI and fluorodeoxyglucose-positron emission tomography (FDG-PET) scans. Independent models for each as well as a combined model for MRI and FDG-PET were developed and used to predict the granular clinical variables. Both MRI and FDG-PET were better at predicting presence of a symptom than severity, suggesting identification of disease state may be more robust than disease stage. FDG-PET predicted predominantly cortical abnormalities better than MRI such as ideomotor apraxia, apraxia of speech and frontal dysexecutive syndrome. MRI demonstrated prediction of cortical and more so sub-cortical abnormalities, such as parkinsonism. Distinct neuroanatomical foci were predictive in MRI- and FDG-PET-based models. For example, vertical gaze palsy was predicted by midbrain atrophy on MRI, but frontal eye field hypometabolism on FDG-PET. Findings also differed by scale or instrument used. For example, prediction of ocular motor abnormalities using the PSP Saccadic Impairment Scale was stronger than with the Movement Disorders Society Diagnostic criteria for PSP oculomotor impairment designation. Combination of MRI and FDG-PET demonstrated enhanced detection of parkinsonism and frontal syndrome presence and apraxia, cognitive impairment and bradykinesia severity. Both MRI and FDG-PET patterns were able to predict some measures in the test set; however, prediction of global cognition measured by Montreal Cognitive Assessment was the strongest. MRI predictions generalized more robustly to the test set. PSP leads to neurodegeneration in motor, cognitive and ocular motor networks at cortical and subcortical foci, leading to diverse yet overlapping clinical syndromes. To advance understanding of phenotypic heterogeneity in PSP, it is essential to consider data-driven approaches to clinical neuroimaging analyses.

## Teaser

Ali *et al*. define data-driven patterns of brain atrophy and hypometabolism across the phenotypic spectrum of progressive supranuclear palsy to capture disease heterogeneity. The relationship between imaging patterns and fine-grained clinical measures illustrates distinct foci of cortical and subcortical brain dysfunction.

## Graphical Abstract

**Graphical Abstract:**
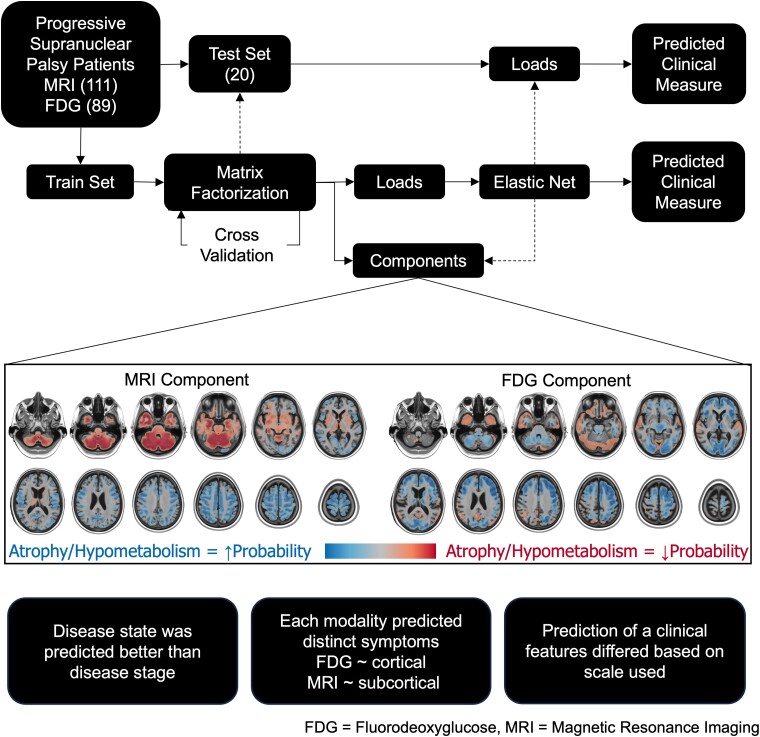

## Introduction

Progressive supranuclear palsy (PSP) is a fatal neurodegenerative tauopathy characterized by neurofibrillary tangles, neuropil threads and astrocytic tau.^1,2^ Phenotypic heterogeneity results from neuropathological change affecting cortical and subcortical regions of motor, cognitive, ocular motor and speech–language networks. The most common existing approach to investigate neurobiological basis of PSP is correlational clinical imaging analyses that start with a clinical scale score or phenotypic label. Such an approach makes the assumptions that the phenotypic label (assigned PSP variant) is accurate and that the measurement instrument adequately captures the inherent disease heterogeneity. The identified brain regions on neuroimaging are then taken as the biological substrate of the deficits. In this study, we present an alternative approach starting with the neuroimaging data and applying data-driven methods to decompose the imaging into distinct patterns—which are taken as reflective of disease heterogeneity—and then determining to what extent these maps onto specific clinical manifestations. With this approach, we will be able to assess whether there are unifying signatures of brain atrophy and hypometabolism that underlie PSP symptoms across variants, whether different patterns are associated with the same symptoms and whether these measurements vary by imaging modality and clinical scale used.

As a relevant background, it is important to discuss sources of heterogeneity that impact findings of clinical neuroimaging analyses in PSP, namely disease biology, measurement instrument and neuroimaging modality. The inherent disease heterogeneity in PSP is illustrated by its many variants. The most common is Richardson syndrome (PSP-RS),^3^ characterized by vertical supranuclear gaze palsy (VSGP), postural instability, frequent falls, axial rigidity and executive dysfunction.^4^ Other variants include frontal,^5^ parkinsonism,^6^ corticobasal syndrome, speech or language disorder,^7^ progressive gait freezing^8^ and postural instability, defined by the Movement Disorders Society Diagnostic criteria for PSP (MDS-PSP criteria).^9^ These cortical and subcortical variants have been differentiated based on structural and neuropathological changes.^10-12^ The criteria also categorize symptoms into four domains (ocular motor, postural instability, akinesia and cognitive).^9^ Patients usually have symptoms within multiple domains, and the most predominant phenotype can change over time.^13,14^ The maximum allocation extinction rules help assign a phenotypic label with the highest level of confidence for PSP neuropathology.^15^ While the reductionist approach may be necessary for many applications, we run the risk of ignoring biologically meaningful clinical variability when deciphering clinical neuroimaging discoveries.^16^ Moreover, while the current diagnosis of PSP is highly sensitive, specificity may be limited for some symptoms and overlap with other tauopathies such as corticobasal degeneration can occur.^13,17^

Another important consideration that motivates our approach is the heterogeneity of clinical scales for similar symptoms. The PSP rating scale aggregates motor, bulbar, cognitive and autonomic symptoms and is a measure of overall disease severity.^18^ The Movement Disorders Society-sponsored revision of the unified Parkinson’s Disease Rating Scale III (MDS-UPDRS III)^19^ quantifies motor symptoms, but individual features, such as parkinsonism, tremor, freezing or postural instability, which may have distinct neural substrates are aggregated into a single score. Aggregation may be less of an issue with domain-specific scales such as the TULIA^20^ test for ideomotor apraxia or the Global Dystonia Rating Scale (GRS),^21^ for example. Grading and definition of motor impairments may also differ between PSP rating scale and MDS-UPDRS III for a given symptom, such as bradykinesia, freezing or postural instability. Similarly, MDS-PSP diagnostic criteria^9^ identify the presence of VSGP, while the PSP Saccadic Impairment Scale (PSIS) defines levels of severity.^22^

**Figure 1:**
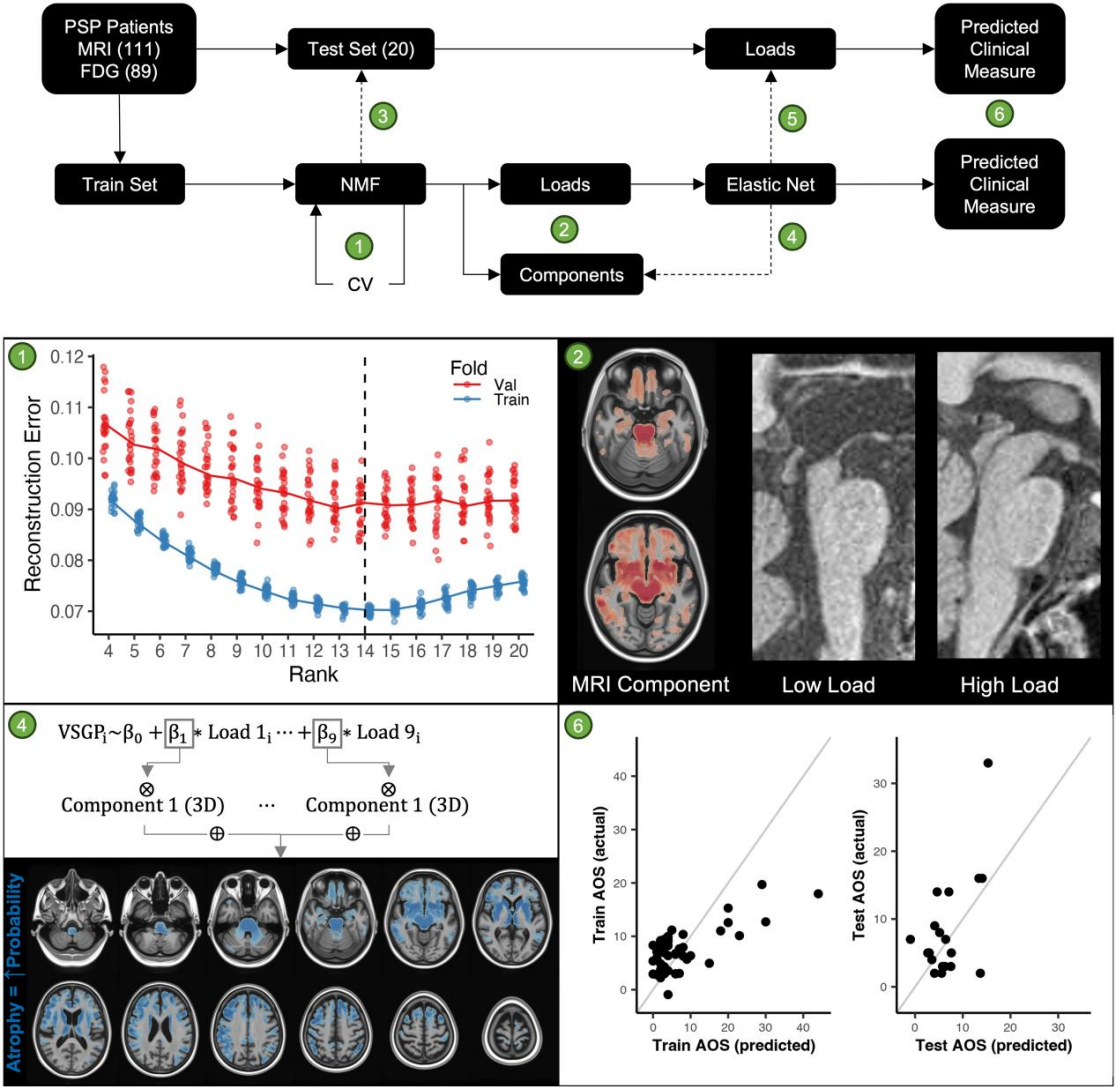
**Overview of our approach with concrete examples of methods.** The top panel shows a process map outlining our methods, with numbers corresponding to the detailed description in this caption. For some numbered items, we have provided concrete examples in the panels below. Starting with the entire cohort, we split off 20 participants with both MRI and FDG available to serve as a test set. We then fit the non-NMF model in the training set, using cross-validation reconstruction error to determine the NMF rank (1). The optimal NMF model is then fit to the training data, resulting in a set of components and participant loads on these components (2). An example of one MRI component is shown (2), with high weights in the midbrain. An example of a participant with a low load and a high load on this component is shown, with low loads corresponding to midbrain atrophy. The NMF model is then applied to the training set (3), resulting in loads for those participants. For each clinical metric of interest, we fit elastic net regression models using loads on all components as predictors (4). To visualize the voxels that contribute the most to a prediction, we create ‘model coefficient maps’ by multiplying the slope of each component load term in the model by the corresponding component weight image and summing these (4). The fitted elastic net models are then applied to the test set (5). For both test and train, the fitted models produce predictions, and these are compared to the true values to assess performance (6). AOS, apraxia of speech; CV, cross-validation; FDG, flouro-deoxy glucose; MRI, magnetic resonance imaging; NMF, non-negative matrix factorization; VSGP, vertical supranuclear gaze palsy.

Finally, neuroimaging modalities may detect difference foci of abnormality within the multi-network degeneration of PSP. Clinical scales or PSP-variant labels serve as a starting point for estimating neuroimaging correlates and determining discriminating imaging findings among patients and controls or among PSP variants. Commonly reported MRI markers include midbrain atrophy; the Magnetic Resonance Parkinsonism Index, which builds upon the midbrain-to-pons ratio; and the most recent Magnetic Resonance Parkinsonism Index 2.0, which includes the third ventricle.^23,24^ In analyses of PSP variants, MRI studies have revealed different patterns of degeneration with cortical versus subcortical predominance in relevant variants which corresponds to regional tau burden.^25^ Fluorodeoxyglucose-positron emission tomography (FDG-PET) has been used to investigate a general pattern of hypometabolism of the cingulate, prefrontal cortex, striatum, thalamus and midbrain (PSP-related pattern).^26,27^ This was used to distinguish PSP from controls and other parkinsonian disorders such as Parkinson’s disease and multiple systems atrophy.^27-29^ Assessment of neurometabolic or neuroanatomic correlates of select PSP symptoms is less common and often focused on a single feature. Frontal lobe hypometabolism on FDG-PET has been associated with cognitive impairment as measured by the mini–mental state examination.^30^ Anterior cingulate hypometabolism was found to correlate with VSGP, while frontal eye field hypometabolism was associated with pursuit impairment.^31^ Hypometabolism in frontal–subthalamic–pedunculopontine loop was found in PSP during walking.^32^ However, no prior studies have explored associations between neuroimaging patterns and granular measures of clinical impairment across the core domains captured by the MDS-PSP criteria and none directly contrasted MRI and FDG-PET. In this article, we perform data-driven analysis of structural atrophy patterns on MRI and brain hypometabolism patterns on FDG-PET. These patterns were used to predict symptom categories across the entire PSP clinical spectrum using granular deconstruction of aggregated clinical scales. Our goal was to assess neuroanatomical underpinnings of clinical features and compare the performance of MRI versus FDG-PET in predicting PSP-related symptoms. We also offer an approach that can be adapted to improve precision of future clinico-radiographic correlative studies. The overall study design is summarized in Fig. 1.

## Materials and methods

### Participants

Patients diagnosed with PSP according to the MDS-PSP diagnostic criteria from two existing cohorts (Dana Foundation and NIH grant R01-NS89757) were included in this study. Patients fulfilling the NINDS-SPSP criteria who were recruited in the Dana Foundation study between 2009 and 2010 and underwent MRI, PET and standardized neurological assessments (F.A., H.B. and K.A.J.), were selected. MDS-PSP criteria were applied retrospectively for these patients. Sporadic PSP patients over the age of 40 recruited into NIH grant R01-NS89757 between 2015 and 2020 fulfilling the MDS-PSP criteria were also selected. All imaging and neurological assessments were completed at the same visit within 2–3 days of each other.

### Standard protocol approvals and patient consent

This study was approved by the Mayo Clinic institutional review board, and written consent was obtained from all participants and/or their qualified representative in keeping with the Declaration of Helsinki.

### Clinical assessments

All patients were evaluated by a neurologist with expertise in neurodegenerative disorders (F.A., K.A.J. and H.B.). Standardized clinical scales used for assessment in PSP were obtained. These included the PSP rating scale,^18^ MDS-UPDRS III,^19^ frontal assessment battery (FAB),^33^ frontal behavioural inventory (FBI),^34^ PSIS,^22^ Montreal Cognitive Assessment (MoCA) battery,^35^ GRS,^21^ Apraxia of Speech Rating Scale (ASRS)^36^ and TULIA screen for apraxia.^20^

Instead of only using the sum score, we selected individual questions within the scales that best represent individual clinical domains such as ocular motor dysfunction, bradykinesia, postural instability, rigidity and tremor (Table 1). Similar clinical domains were derived from the remaining scales. Binary outcomes were described as the presence or absence of a given clinical symptom. The total scores and subscores were used as a marker of symptom severity. Since no patients had rest, postural or kinetic tremor, these were omitted from all analyses.

**Table 1:** Clinical scales and assessed features

Clinical domain	Outcome type	Clinical scale/criteria used	Assessed items
Parkinsonism	Binary	MDS-PSP^a^ A2, A3	
Bradykinesia	Severity	PSPRS MDS-UPDRS	20, 21 3.4–3.8
Rigidity	Severity	PSPRS	18
		MDS-UPDRS	3.3
Tremor	Severity	PSPRS	23
		MDS-UPDRS	3.15–3.18
Apraxia	Binary	PSPRS	22
	Severity	TULIA	Total score
Dystonia	Binary	GRS/PSPRS	19
		GRS	Total score
Dysarthria	Binary	Yes/no	
	Severity	Dysarthria rating scale	0–4
Apraxia of speech	Binary	Yes/no	
	Severity	ASRS	Total score
Language dysfunction	Binary	MDS-PSP^a^ C3	
Cognitive	Severity	MoCA	
Frontal syndrome	Binary	MDS-PSP C2^a^	
	Severity	FAB, FBI	Total score
VSGP	Binary	MDS-PSP O1^a^, PSIS	
	Severity	PSPRS	14–16
Postural instability	Binary	MDS-PSP P1–P3^a^	
	Severity	PSPRS	5, 27
		MDS-UPDRS	3.12
Gait freezing	Binary	MDS-PSP A1^a^	
		MDS-UPDRS	3.11
Gait	Severity	PSPRS	26

Clinical domains were assessed using binary outcome (present/absent), total score of the scale and select questions from clinical scales were used to assess individual symptoms and their severity; FAB, frontal assessment battery; FBI, frontal behavioural inventory; GRS, Global Dystonia Rating Scale; MoCA, Montreal Cognitive Assessment; MDS-UPDRS, Movement Disorders Society-sponsored revision of the unified Parkinson’s Disease Rating Scale; MDS-PSP, Movement Disorders Society PSP Diagnostic criteria; PSPRS, PSP Rating Scale.

^a^Clinical feature as defined by the MDS diagnostic criteria for PSP.

### Image acquisition

All participants had 3-T MRI performed on a Siemens Prisma or GE scanner (GE Healthcare, Chicago, IL, USA) using a standardized protocol that can be found in our previous publications.^37-39^ In brief, 3D magnetization prepared rapid acquisition gradient echo (MPRAGE) sequence was obtained with the same parameters on a Siemens Prisma or GE scanner: repetition time/echo time/inversion time: 2300/3/900 ms; flip angle 8°; 26 cm field of view; 256 × 256 in-plane matrix with a phase field of view of 0.94 and slice thickness of 1.2 mm, in-plane resolution 1. Scanner type was not found to impact analyses or results (detailed assessment is found in Supplementary Materials). All MPRAGE images were corrected for gradient non-linearity and intensity non-uniformity.

A subset of participants underwent fluorine 18 (F-18) FDG-PET imaging performed using an identical standardized protocol on the same PET/CT scanner (GE Healthcare) operating in 3D mode. Fluorodeoxyglucose (F18), 459 MBq (range 367–576 MBq), was injected intravenously in a dimly lit room, and after a 30-min uptake period, an 8-min FDG scan was performed consisting of four 2-min dynamic frames following a low-dose CT transmission scan. Standard corrections were applied to address attenuation, scatter, random coincidences and decay. The four-frame sequences were summed to create a single static image.

### Image processing

Details of our fully automated pipeline using advanced normalization tools^40^ and SPM12^41^ with in-house modifications and tools have been described previously.^42,43^ MPRAGE scans were normalized to the Mayo Clinic Adult Lifespan Template (MCALT) and segmented with MCALT priors/settings.^44^ The grey matter and white matter probability maps from the segmentation were combined into tissue probability maps for each participant. The spatial normalization parameters were then used to propagate these tissue probability maps to MCALT space, where they were smoothed at 6 mm full width at half-maximum prior to further MRI analyses. For participants with PET available, the images were co-registered to the associated MPRAGE. The template-to-participant mapping from the MRI segmentation was then used to propagate the automated anatomical lebeling AAL atlas to participant space.^45,46^ The median uptake value in the eroded supratentorial white matter was calculated, and every voxel in the FDG-PET was divided by this value to obtain an FDG-PET standardized uptake value ratio image. These standardized uptake value ratio images were then warped to MCALT space using the MRI normalization parameters and smoothed at 6 mm full width at half-maximum prior to further PET analyses. Both the MRI tissue volume and the PET images were masked using a dilated grey matter mask from MCALT, with the addition of posterior fossa white matter voxels, to focus on voxels with high likelihood of capturing PSP pathophysiology. Adequately capturing brainstem areas typically affected by PSP, such as the midbrain volume, requires inclusion of white matter voxels in the region. Indeed, prior studies have shown white matter loss in the brainstem in PSP.^22,25^

### Non-negative matrix factorization

The overall study approach is summarized in Fig. 1. Non-negative matrix factorization (NMF) is a dimensionality reduction technique that applies to strictly positive data. Like other matrix factorization techniques, such as principal component analysis or independent component analysis, it takes as input a subject (row)-by-feature (column) matrix and produces two new matrices: a subject (row)-by-component (column) matrix and a component (row)-by-feature (column) matrix. The matrix product of these two approximates the input matrix. When applied to neuroimaging, the features are typically voxel values on the applicable modality, and the factorization process involves identifying a low-dimensional (rank) set of patterns in the voxel data that can explain a lot of the between-subject variation. What makes NMF different from other techniques is that the subject-load and voxel-weight matrices are constrained to only contain positive values. This aids interpretability since it avoids the interaction of positive and negative subject-loads with positive and negative voxel weights. However, it can complicate the computation of the components and the weights, since it involves decomposing a matrix into elements, which can now combine in a strictly additive manner (i.e. less flexibility).

Several algorithms for NMF exist, each with their own assumptions as to the nature of the input data. We chose the ‘minimum-volume rank-deficient’ algorithm^47^ implemented in MATLAB^48^ with slight modifications for our use case. This algorithm has recently been shown to be robust and results in a unique solution for a given input matrix.^47^ It has also been used successfully in related domains of multispectral satellite image segmentation and audio source separation, where it had favourable qualities over other NMF approaches.^47,49^ Specifically, it works in rank-deficient cases, which is likely true for neuroimaging data given the high degree of smoothing and spatial covariance. The only tunable parameter is the rank, or the number of components, to identify. We determined this via 5-fold cross-validation in the training set. This is premised on the idea that a good choice of rank should satisfy the following: (i) the reconstruction error should be low in the data set used to find the patterns (i.e. the key sources of variation are accounted for) and (ii) the reconstruction error in an out of sample data set should not be inflated (i.e. we have not overfit the discovery data set by finding unique or noise components). If the rank is under- or over-estimated, then (i) may not be satisfied, whereas overfitting the ascertainment data set would result in (ii) not being satisfied. As such, we took the training set and divided it into 5 equal folds. We then ran the NMF algorithm on 4 of the folds and reconstructed those data and then applied the fitted model to the 1 left-out fold and reconstructed those data. The reconstruction error (mean absolute error) was then calculated for both the discovery and the hold-out fold. This was repeated five times—with each fold serving as the hold-out fold once. Because the fold splits are random, we repeated the entire cross-validation process five times, too. The result is that, for any given rank, there were 25 pairs of discovery and hold-out reconstruction errors. We completed this procedure for ranks 4 through 20 and then examined the discovery and hold-out losses, with the goal of choosing the rank for which both were at or near their lowest point. We then computed the NMF solution for the entire training data set at this rank. The voxel-weight matrix from the training data was then used to obtain loads for the test set using the same NMF algorithm. The resulting participant component loads (test and train) were then used in further statistical analyses, while the voxel-weight matrix was used to visualize the components or patterns that were identified.

### Statistical analyses

All statistical analyses were performed in R version 4.05. We used the *glmnet* package to fit penalized regression (elastic net) models where the clinical variable or score of interest was the outcome, and age and component loads were the predictors.^50^ The elastic net penalty, denoted by alpha in *glmnet*, controls the balance of L2 (ridge) and L1 (lasso) regularization. It was set to 0.2, i.e. favouring L2 regularization, for individual modality modals and 0.8, i.e. favouring L1, for combined FDG-PET and MRI models. The lambda parameter, which sets the strength of the regularization, was optimized using 5-fold cross-validation in the training set. The result is a sparse solution with shrunken coefficient estimates. Separate models were fit in the training set for each clinical outcome of interest and for each modality. These fitted models were then used to predict the clinical outcome in the test set as a true out of sample validation of the clinical information captured in the MRI and FDG-PET components. For clinical outcomes that were continuous, we used a linear design, whereas binary outcomes were modelled using logistic forms of elastic net regression. To summarize the performance of each model, we calculated the *R*^2^ for continuous outcomes and the Matthews correlation coefficient (MCC) and accuracy for binary outcomes. Our focus was on the MCC given its desirable properties for binary classification tasks with imbalanced data sets, but we report both.^51^ Specifically, the MCC ranges from −1 to 1, with 0 being the expected performance for a ‘coin-tossing classifier’ and higher values indicating models that can correctly predict most positive data instances and most negative data instances.

**Figure 2:**
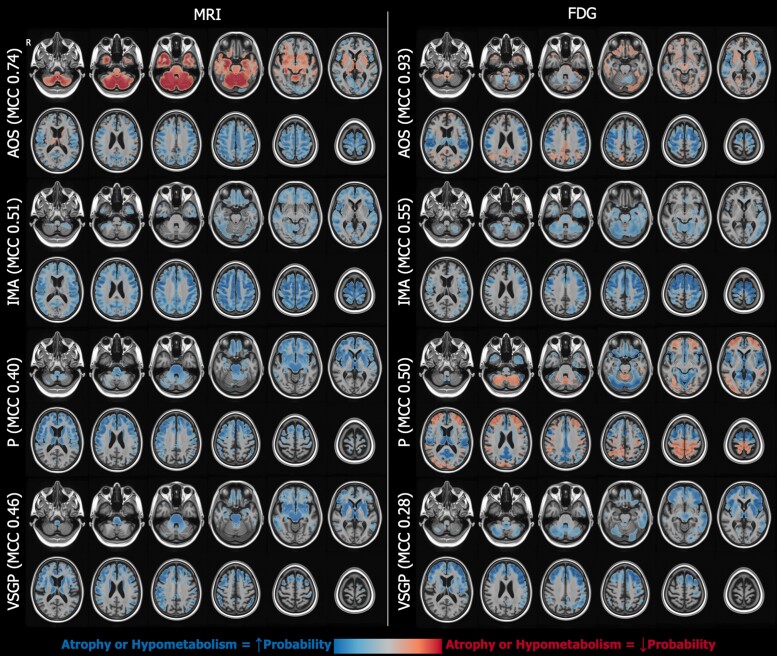
**Voxel-level illustration of model beta weights for binary variables present.** Estimates for the MRI models are shown on the left and FDG-based models on the right. Blue voxels represent those where increased atrophy/hypometabolism was associated with a higher probability of the binary clinical feature being present. Frontal cortical involvement was associated with AOS and IMA, with FDG models showing more regional specificity—ventrolateral precentral for AOS and dorsolateral for IMA. Subcortical involvement was associated with a lower probability of AOS, possibly reflecting cortical–subcortical phenotypic variability as discussed in the text. Frontal cortical and subcortical, as well as brainstem, atrophy was associated with parkinsonism and VSGP for MRI models. For FDG models, the pattern for parkinsonism was less clear—anterior frontal and superior parietal involvement was associated with a lower probability of parkinsonism, whereas precentral and superior frontal areas were associated with increased probability. The FDG association for presence of VSGP was similar to that of MRI, but with more cortical and less subcortical weighting. AOS, apraxia of speech; FDG, flouro-deoxy glucose; MCC, Matthews correlation coefficient; IMA, ideomotor apraxia; P, parkinsonism; VSGP, vertical supranuclear gaze palsy.

**Figure 3:**
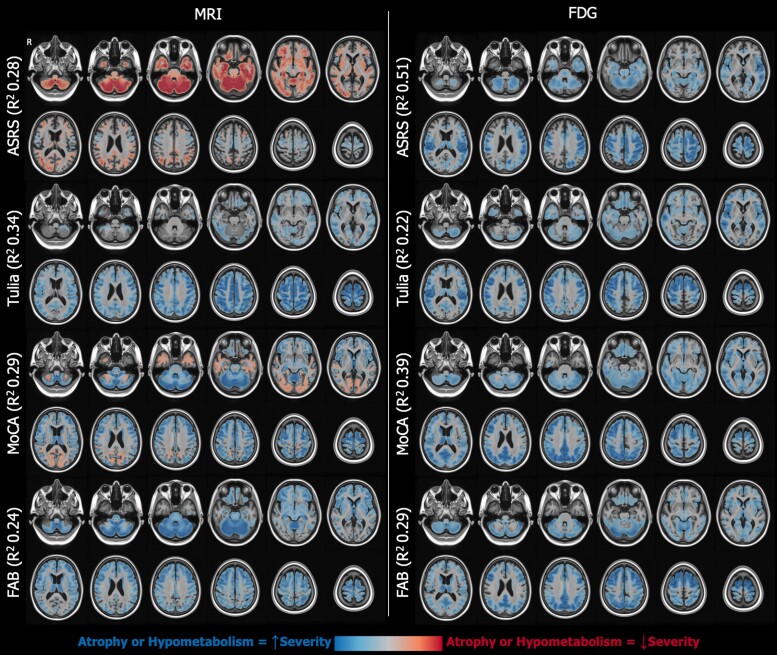
**Voxel-level illustration of model beta weights for select continuous variables.** Estimates for the MRI models are shown on the left and FDG-based models on the right. Blue voxels represent those where increased atrophy/hypometabolism was associated with a higher predicted severity on the clinical feature, whereas red voxels indicate those where atrophy/hypometabolism was associated with a lower predicted severity. MRI- and FDG-based models both associated motor and premotor atrophy/hypometabolism with increased severity, but MRI additionally had widespread inverse associations, including subcortical and cerebellar atrophy being associated with less severe ASRS. This may reflect a cortical versus subcortical phenotype effect. For TULIA and MoCA, in contrast, the regions associated with increased severity were similar. Interestingly, although both MRI and FDG associated frontal hypometabolism/atrophy with worse performance on the FAB, the MRI models placed more emphasis on cerebellar voxels and the FDG models more on parietal voxels. ASRS, Apraxia of Speech Rating Scale; FAB, frontal assessment battery; FDG, fluorodeoxyglucose; MoCA, Montreal Cognitive Assessment; MRI, magnetic resonance imaging; TULIA, apraxia scale.

To enhance interpretability of our models, we also projected the coefficients from the penalized models back into voxel space. This is possible since we used linear models with a coefficient for each of the imaging components included in the model, and so, it is possible to propagate the estimated slopes for each component back to voxel space. After multiplying the voxel-level weight map for each component by the respective slope for that component in the respective model, we added the resulting images together to get a single 3D image. These model coefficient maps show the magnitude and the direction of contribution of each voxel to the prediction by the respective model, e.g. whether atrophy or hypometabolism in a brain region increased or decreased the probability for the presence of a gaze palsy (Figs. 2 and 3 detailed in Results section).

## Results

### Cohort

The entire cohort comprised 111 patients with PSP. Ninety-one patients (47% women, median age 70.8 years, median disease duration 4.3 years) were used as the training set. All had MRI, and 69 had FDG-PET available. Among the training set, 49 had Richardson syndrome (PSP-RS) and 42 had variant PSP phenotypes (24 were cortical such as frontal, corticobasal syndrome and speech/language variants and 18 were subcortical such as parkinsonism and gait freezing). Twenty patients (45% women, median age 69 years, median disease duration 4.4 years) were used as the test set. All had MRI and FDG-PET available. Among the test set, 11 had Richardson syndrome and 9 had variant PSP phenotypes (5 cortical and 4 subcortical). Due to a limited number of variant PSP syndromes, stratification by syndrome or using variant syndromes as an outcome could not be undertaken. Clinical features derived from scales are described in Table 1.

### Imaging components

The optimal NMF for MRI and FDG-PET data was determined to be at a dimensionality of 9 and components and 14 components, respectively. Figures showing the rationale for selecting these ranks are shown in the Supplementary Section. Examples are shown in Figs. 2 and 3, along with participant-level images and loads to enhance interpretability. Findings will be detailed below. The areas of maximal voxel weights for all individual MRI and FDG-PET components are shown in the Supplementary Section.

### MRI and fluorodeoxyglucose prediction of binary outcomes

MRI atrophy and FDG-PET hypometabolism patterns were used to predict the presence of a clinical feature (Table 2). Overall, imaging patterns on both MRI and FDG-PET performed well at predicting a binary outcome (the presence of a clinical symptom).

**Table 2:** MRI and FDG-PET prediction of binary outcomes

	MRI				FDG						
Measure	*N*	Symptom present (*n*)	MCC	Accuracy	*N*	Symptom present (*n*)	MCC	Accuracy	Error MRI	Error FDG	*P*
Dysarthria	55	50	0.169	0.673	50	45	0.903	0.980	0.320	0.020	0.001**^,a^
Dystonia (GRS)	56	20	0.224	0.607	51	15	0.159	0.608	0.392	0.392	1.000
Dystonia (PSPRS)	83	16	0.366	0.711	64	8	0.440	0.766	0.234	0.234	1.000
Freezing (MDS)	86	9	0.228	0.686	66	9	0.843	0.955	0.273	0.045	0.001**^,a^
Frontal syndrome (MDS)	86	20	0.216	0.640	66	13	0.437	0.758	0.364	0.242	0.186
Limb apraxia (PSPRS)	80	27	0.509	0.762	63	18	0.550	0.810	0.194	0.177	1.000
Parkinsonism (MDS)	86	75	0.403	0.756	66	56	0.500	0.818	0.258	0.182	0.332
Postural instability	86	75	0.377	0.733	66	56	0.239	0.697	0.273	0.303	0.838
Speech apraxia	57	8	0.743	0.912	51	8	0.932	0.980	0.098	0.020	0.134
Speech–language (MDS)	86	15	0.330	0.698	66	13	0.497	0.773	0.273	0.227	0.663
VSGP (MDS)	86	60	0.451	0.733	66	37	0.286	0.652	0.273	0.348	0.486
VSGP (PSIS)	86	54	0.460	0.744	67	35	0.280	0.642	0.231	0.354	0.230

GRS, Global Dystonia Rating Scale; MDS, Movement Disorders Society criteria for PSP; MCC, Mathews correlation coefficient; PSPRS, PSP Rating Scale; VSGP, vertical supranuclear gaze palsy.

^a^FDG outperforms MRI.

**significant after correction for multiple comparisons.

FDG-PET models effectively predicted dysarthria, gait freezing, speech apraxia and limb apraxia, with MCC values ranging from 0.8 to 0.9. Good prediction was also found for dystonia as measured by PSP rating scale, parkinsonism, speech–language dysfunction, and frontal syndrome with MCC values of 0.4–0.55. MRI patterns demonstrated good prediction of limb and speech apraxia (MCC 0.7 and 0.5) and reasonable prediction of VSGP, parkinsonism, postural instability and dystonia (MCC 0.4–0.5).

Upon comparing relative performance of MRI and FDG-PET, statistically significant differences were only observed in the prediction accuracy of dysarthria and gait freezing, where FDG-PET outperformed MRI (*P* < 0.001). MRI performed marginally better than FDG-PET in predicting presence of VSGP and postural instability, but this was not statistically significant.

Findings varied by clinical scale used. For example, predicting the presence of VSGP was stronger as denoted by PSIS compared to MDS-PSP criteria. Similarly, predicting presence of dystonia by either MRI or FDG-PET was stronger using PSP rating scale (MCC 0.37 and 0.44) compared to GRS (MCC 0.22 and 0.16).

As shown in Fig. 2, an anatomical substrate of the predicted symptoms differed. MRI atrophy primarily in subcortical regions such as the midbrain predicted VSGP, while on FDG-PET hypometabolism in cortical regions such as the frontal eye field predicted VSGP. Basal ganglia atrophy on MRI predicted the presence of parkinsonism, while that on FDG-PET motor and premotor cortical hypometabolism predicted parkinsonism. Gait freezing corresponded with sub-cortical, midbrain and frontal atrophy and hypometabolism. Ideomotor apraxia and apraxia of speech correlated with atrophy and hypometabolism in frontoparietal cortical regions.

### MRI and fluorodeoxyglucose prediction of continuous outcomes

MRI atrophy and FDG-PET hypometabolism patterns were used to predict clinical disease severity (Table 3). Clinical scale and subsection scores (as described in Table 1) that define overall disease or symptom specific severity were used.

**Table 3:** MRI and FDG-PET prediction of symptom severity

	MRI				FDG				Comparison		
Measure	*N*	Median	IQR	*R* ^2^	*N*	Median	IQR	*R* ^2^	MAE (MRI)	MAE (FDG)	*P*-value
ASRS total	50	4.0	6.00	0.282	48	4.0	6.00	0.506	3.875	4.340	0.257
Behavioural inventory (FBI)	51	40.0	41.00	0.223	44	40.0	41.50	0.044	15.984	18.750	0.055
Bradykinesia severity (PSPRS)	82	2.0	2.00	0.195	63	2.0	2.00	0.255	0.728	0.625	0.240
Bradykinesia severity (UPDRS)	87	24.0	13.50	0.255	67	21.0	15.50	0.252	4.892	6.321	0.943
Dysarthria severity	50	2.0	1.50	0.009	45	2.0	1.50	0.034	0.954	0.717	0.559
Eyes down (PSPRS)	83	3.0	2.00	0.075	64	3.0	3.00	0.227	1.119	1.136	0.761
Eyes Horizontal (PSPRS)	83	1.0	1.50	0.160	64	1.0	2.00	0.026	0.785	0.660	0.112
Eyes total (PSPRS)	83	8.0	5.50	0.099	64	7.5	6.00	0.223	2.856	2.801	0.506
Eyes vertical (PSPRS)	83	7.0	4.00	0.041	64	6.0	4.25	0.202	2.001	1.940	0.308
FAB total	85	14.0	4.00	0.241	67	14.0	4.00	0.286	1.830	1.209	0.103
Gait severity (PSPRS)	83	3.0	1.00	0.328	64	3.0	2.00	0.290	0.625	0.700	0.424
MoCA	56	23.0	7.00	0.287	51	23.0	6.50	0.390	2.747	2.001	0.021*^,a^
Postural severity (PSPRS)	83	6.0	2.00	0.241	64	5.0	3.00	0.204	1.275	1.366	0.771
Postural severity (UPDRS)	87	3.0	1.00	0.263	67	3.0	1.00	0.371	0.718	0.551	0.252
PSPRS total score	82	42.0	19.50	0.216	64	37.0	17.75	0.228	9.688	8.900	0.470
Rigidity appendicular (PSPRS)	83	1.0	1.50	0.195	64	1.0	2.00	0.090	0.752	0.922	0.090
Rigidity appendicular (UPDRS)	87	4.0	7.00	0.203	67	4.0	6.50	0.150	2.350	2.850	0.085
Rigidity axial (PSPRS)	83	2.0	2.00	0.228	64	2.0	1.00	0.025	0.645	0.674	0.056
Rigidity axial (UPDRS)	87	3.0	2.00	0.247	67	2.0	2.00	0.235	1.015	1.026	0.918
Rigidity total (PSPRS)	83	4.0	3.00	0.279	64	2.5	3.00	0.142	1.121	1.354	0.238
Rigidity total (UPDRS)	87	6.0	7.00	0.253	67	4.0	7.00	0.329	3.131	2.752	0.232
TULIA praxis	54	11.0	3.75	0.336	46	11.0	2.75	0.217	1.610	1.107	0.020*^,a^
UPDRS III	88	47.5	25.00	0.302	68	39.0	23.50	0.203	10.091	10.294	0.397

^a^FDG outperforms MRI.

^*^Significant prior to correction for multiple comparisons.

Overall disease severity, as measured by UPDRS III and PSP rating scale, was predicted modestly by both MRI and FDG-PET (*R*^2^ = 0.2–0.3). Prediction of severity of individual clinical features was overall modest when compared with binary presence/absence as described above. FDG-PET prediction was the strongest for apraxia of speech severity (*R*^2^ = 0.50), global cognition measured by MoCA (*R*^2^ = 0.39), postural instability (*R*^2^ = 0.37) and parkinsonism as defined by bradykinesia and rigidity subscores (*R*^2^ = 0.25–0.33). While binary presence of limb apraxia was predicted well (MCC = 0.5), severity measured by TULIA was more challenging to predict (*R*^2^ = 0.22 for MRI and 0.34 for FDG-PET).

MRI prediction of symptom severity was modest with *R*^2^ values ranging from 0.2 to 0.34 for parkinsonism (bradykinesia and rigidity), postural instability, gait impairment, global cognition, limb and speech apraxia.

Strength of prediction of the clinical severity also differed by measurement instrument or scale. For example, prediction of postural instability, as measured by UPDRS III, was better than the measures in the PSP rating scale, and prediction of frontal dysexecutive syndrome as measured by FAB was stronger than as measured by the FBI.

FDG-PET was statistically significantly better than MRI in predicting MoCA score (median absolute error [MAE] 2.0 versus 2.8, *P* = 0.02) and TULIA score for limb apraxia (MAE 1.1 versus 1.6, *P* = 0.02). In all other cases, the MAE was comparable between MRI and FDG-PET.

Figure 2 shows atrophy and hypometabolism patterns corresponding with symptom severity. Frontal, cortical predominant hypometabolism on FDG-PET predicted apraxia of speech, limb apraxia (TULIA) and cognitive dysfunction (MoCA and FAB). A similar but less robust signal was seen on MRI, with frontal cortical atrophy associated with the severity of these symptoms. A lower probability of subcortical atrophy on MRI (Fig. 2, top left) was associated with apraxia of speech severity, demonstrating a cortical versus subcortical syndrome divide. Frontal syndrome severity was predominantly associated with frontal cortical atrophy; however, the pattern also included midbrain atrophy, possibly pointing to overall disease severity.

### MRI, fluorodeoxyglucose versus combination of both in predicting binary outcomes

Combination of the MRI and FDG models predicted the presence of most clinical features as evidenced by high MCC values (Table 4). The combination model was statistically significantly better than either MRI or FDG-PET model alone for the presence of parkinsonism and frontal syndrome. The combination model also performed better (higher MCC, lower error) in predicting presence of dystonia, limb apraxia and speech apraxia, although this was not statistically significant. FDG-PET was significantly better than the combination model in predicting presence of gait freezing.

**Table 4:** MRI, FDG and combination of both modalities in predicting binary and continuous clinical symptoms

	Combination					Versus MRI		Versus FDG	
Binary measure	*N*	Present	MCC	Accuracy	Error	Error	*P*-value	Error	*P*-value
Dysarthria present	50	45	1.000	1.000	0	0.320	0.001	0.020	1.00
Dystonia present (GRS)	51	15	0.244	0.667	0.333	0.392	0.663	0.392	0.546
Dystonia present (PSPRS)	64	8	0.541	0.797	0.203	0.234	0.789	0.234	0.773
Freezing present (MDS)	66	9	0.625	0.848	0.152	0.273	0.080	0.045	0.023*^,a^
FTD-syndrome (MDS)	66	13	1.000	1.000	0	0.364	<0.001**^,b^	0.242	0.001**^,b^
Limb apraxia present (PSPRS)	63	18	0.708	0.857	0.129	0.194	0.386	0.190	0.546
Parkinsonism present (MDS)	66	56	1.000	1.000	0	0.258	<0.001**^,b^	0.182	0.006**^,b^
Postural present (MDS)	66	56	0.273	0.727	0.273	0.273	1.000	0.303	0.803
Speech apraxia present	51	8	0.873	0.961	0.039	0.098	0.248	0.020	1.000
Speech–language present (MDS)	66	13	0.437	0.758	0.242	0.273	0.773	0.227	1.000
VSGP present (MDS)	66	37	0.290	0.652	0.348	0.273	0.458	0.348	1.000
VSGP present (PSIS)	67	35	0.220	0.612	0.385	0.231	0.024	0.358	0.838

	Combination					Versus MRI		Versus FDG	
Continuous measure	*N*	Median	IQR	*R* ^2^	MAE	MAE	*P*-value	MAE	*P*-value
ASRS total	48	4.0	6.00	0.426	4.714	3.875	0.376	4.271	0.186
Behavioural inventory (FBI)	44	40.0	41.50	0.064	19.191	15.984	0.097	18.750	0.551
Bradykinesia severity (PSPRS)	63	2.0	2.00	0.085	0.623	0.728	0.103	0.625	<0.001**^,b^
Bradykinesia severity (UPDRS)	67	21.0	15.50	0.097	7.019	4.892	0.008**^,c^	6.321	0.002**^,a^
Dysarthria severity	45	2.0	1.50	0.082	0.702	0.954	0.268	0.768	0.143
Eyes down (PSPRS)	64	3.0	3.00	0.183	1.212	1.119	0.933	1.136	0.144
Eyes horizontal (PSPRS)	64	1.0	2.00	0.069	0.671	0.785	0.165	0.660	0.132
Eyes total (PSPRS)	64	7.5	6.00	0.152	2.911	2.856	0.652	2.801	0.068
Eyes vertical (PSPRS)	64	6.0	4.25	0.082	2.229	2.001	0.238	1.940	0.003**^,a^
FAB total	67	14.0	4.00	0.256	1.412	1.830	0.151	1.201	0.088
Gait severity (PSPRS)	64	3.0	2.00	0.059	0.707	0.625	0.001**^,c^	0.700	<0.001**^,a^
MoCA	51	23.0	6.50	0.415	1.681	2.747	0.005**^,b^	2.221	0.870
Postural severity (PSPRS)	64	5.0	3.00	0.063	1.285	1.275	0.155	1.366	0.066
Postural severity (UPDRS)	67	3.0	1.00	0.320	0.663	0.718	0.530	0.551	0.037*^,a^
PSPRS total score	64	37.0	17.75	0.180	8.743	9.688	0.888	8.971	0.031*^,b^
Rigidity appendicular (PSPRS)	64	1.0	2.00	0.131	0.890	0.752	0.146	0.922	0.099
Rigidity appendicular (UPDRS)	67	4.0	6.50	0.109	3.073	2.350	0.010**^,c^	2.850	0.048*^,a^
Rigidity axial (PSPRS)	64	2.0	1.00	0.048	0.688	0.645	0.102	0.674	0.020*^,a^
Rigidity axial (UPDRS)	67	2.0	2.00	0.265	0.919	1.015	0.526	1.026	0.474
Rigidity total (PSPRS)	64	2.5	3.00	0.104	1.242	1.121	0.078	1.354	0.063
Rigidity total (UPDRS)	67	4.0	7.00	0.131	3.152	3.131	0.026*^,c^	2.752	<0.001**^,a^
TULIA praxis	46	11.0	2.75	0.289	1.047	1.610	0.003**^,b^	1.101	0.282
UPDRS III	68	39.0	23.50	0.141	11.627	10.091	0.159	10.380	0.009**^,a^

^*^Significant prior to correction for multiple comparisons; **significant after correction for multiple comparisons using false discovery rate. ^a^FDG outperforms combination; ^b^Combination outperforms single modality; ^c^MRI outperforms combination.

### MRI, fluorodeoxyglucose and combination of both in predicting continuous outcomes

The clinical symptom severity was predicted using the combination of MRI and FDG-PET and compared to individual modalities (Table 4). The combination was statistically significantly better at predicting clinical severity of bradykinesia measured by PSP rating scale (*P* = 0.001), cognitive impairment measured by the MoCA (*P* = 0.005) and limb apraxia measured by TULIA (*P* = 0.003).

MRI and FDG-PET both performed significantly better on their own than the combination in predicting bradykinesia measured by the UPDRS, appendicular rigidity and gait impairment. FDG-PET performed significantly better than the combination in predicting total UPDRS III score, PSP rating scale score, postural instability and VSGP.

### Predictive performance in a test set

In a hold-out set of 20 patients, who were not used in training the NMF or the predictive models, we applied the MRI, FDG-PET and combination models to predict presence of a symptom and its severity (Table 5). MRI was able to predict presence of speech apraxia, limb apraxia, frontal syndrome and gait freezing well. FDG-PET predicted limb apraxia, speech apraxia, frontal syndrome and VSGP. The combination of both modalities was not clearly superior in any scenario.

**Table 5:** Predictive performance in test set

			MRI		FDG		Combo	
Binary measure	*N*	Present	MCC	Accuracy	MCC	Accuracy	MCC	Accuracy
Dysarthria present	19	18	0.160	0.37	−0.056	0.9	−0.056	0.9
Dystonia present (PSPRS)	20	5	−0.067	0.6	−0.378	0.45	−0.333	0.5
Freezing present (MDS)	20	4	0.375	0.8	0.062	0.7	0.140	0.75
FTD-syndrome (MDS)	20	6	0.312	0.6	0.218	0.6	0.285	0.65
Limb apraxia present (PSPRS)	20	7	0.341	0.7	0.471	0.75	0.206	0.65
Postural present (MDS)	20	17	0.140	0.75	−0.380	0.4	−0.275	0.55
Speech apraxia present	20	2	0.667	0.9	0.250	0.8	0.250	0.8
Speech–language present (MDS)	20	2	0.105	0.65	0.145	0.7	0.250	0.8
VSGP present (MDS)	20	17	0.140	0.75	0.140	0.55	0.229	0.65
VSGP present (PSIS)	20	16	0.000	0.65	0.408	0.6	−0.105	0.55

Continuous measure	*R* ^2^ (MRI)	*R* ^2^ (FDG)	*R* ^2^ (Combo)
ASRS total	0.359	0.274	0.439
Behavioural inventory (FBI)	0.037	0.028	0.043
Bradykinesia severity (PSPRS)	0.019	0.094	0.043
Bradykinesia severity (UPDRS)	0.000	0.062	0.056
Eyes down (PSPRS)	0.000	0.169	0.008
Eyes total (PSPRS)	0.000	0.095	0.000
Eyes vertical (PSPRS)	0.000	0.052	0.000
FAB total	0.245	0.196	0.177
MoCA	0.423	0.471	0.482
Postural severity (UPDRS)	0.082	0.000	0.000
Rigidity axial (PSPRS)	0.028	0.000	0.012
Rigidity axial (UPDRS)	0.000	0.092	0.016
Rigidity total (PSPRS)	0.030	0.044	0.026
Tulia praxis	0.203	0.156	0.181
UPDRS III	0.007	0.006	0.004

The following were not analysed in the test set as performance in the training set was poor (MCC < 0.25, *R*^2^ < 0.2): dystonia present (GRS), dysarthria severity, eyes horizontal (PSPRS) and rigidity appendicular (PSPRS). Not shown in the table are the following for which the *R*^2^ in the test set was zero for all three models: rigidity appendicular (UPDRS), postural severity (PSPRS), gait severity (PSPRS), dystonia rating scale total (GRS), PSPRS total score and rigidity total (UPDRS).

For continuous measures of disease severity, combination of MRI and FDG-PET predicted ASRS score (*R*^2^ = 0.44) and MoCA (*R*^2^ = 0.48) better than either modality alone. MRI appeared to generalize to the test set better than FDG-PET albeit by a small margin in predicting severity of apraxia of speech, limb apraxia and frontal syndrome. Both MRI and FDG-PET generalized most strongly for prediction of MoCA score in the test set, likely since it reflects global cognitive dysfunction.

The test set findings also serve to illustrate the difference between accuracy and MCC. We can consider VSGP measured using PSIS as an example. Both MRI and FDG have an accuracy of around 0.6. However, in the case of MRI the model correctly predicted VSGP in 12/16 patients and incorrectly predicted it as being present in 3/4 of patients who did not have it. For FDG, 0/4 of the patients without VSGP were predicted as having it, whereas it was correctly predicted in 8/16 who had VSGP. MCC penalizes misclassifications of the minor or less common class, in this case the absence of VSGP.

## Discussion

In this article, we present data-driven comparative analysis of MRI and FDG-PET patterns and how they predict presence and severity of clinical features across the PSP spectrum. This is the first data-driven comparative analysis of both MRI and FDG-PET where we describe patterns of atrophy and hypometabolism across the PSP spectrum. We used aggregate clinical scale scores as well as fine-grained assessment of presence and severity of individual symptoms (tremor, rigidity, bradykinesia, frontal dysexecutive, apraxia, dysarthria, VSGP, etc.). Key findings include (i) models predicting the presence/absence of PSP features, performed better than models predicting severity of a feature; (ii) FDG-PET patterns predicted cortical features better while MRI predicted subcortical features better; (iii) strength of prediction of a clinical feature varied based on measurement scale; (iv) MRI and FDG-PET may reveal distinct neuroanatomical foci underlying a PSP clinical feature; and (v) predictive performance in a hold-out set showed reduced performance, with the best generalizability for cortically based measures (e.g. cognitive impairment measured by the MoCA).

We include PSP Richardson syndrome, as well as variant phenotypes of PSP, whereas previous data-driven analyses of neuroimaging in PSP have been primarily used to differentiate PSP from other parkinsonian disorders based on patterns of frontal, basal ganglia and midbrain hypometabolism.^29,52^ A prior principal component analysis was used to assess 310 patients with frontotemporal lobar degeneration syndromes; the report concluded that syndromes did not fit mutually exclusive categories rather existed on a continuum in a multi-dimensional space depending on neural networks affected, which is consistent with our findings.^53^ Most recently, a data-driven evaluation of a large cohort was used to describe cortical and subcortical disease states in PSP, but association with fine-grained clinical metrics has not been performed previously.^54^

In our study, patterns seen on MRI and FDG-PET appeared to capture a cortical or subcortical predominance of impairment. Our findings support the neuropathological pattern described by Kovacs *et al.*^11^ as well as recent findings by Scotton *et al.*^12,54^, both of whom showed a predominantly cortical versus subcortical pattern of progression in PSP. While both MRI and FDG-PET patterns predicted a variety of symptoms in our cohort, valuable insights can be gained from where they differed. MRI pattern of atrophy in basal ganglia and midbrain appeared to outperform FDG-PET in prediction of subcortical deficits such as parkinsonism and gait as measured by the PSP rating scale. In contrast, frontoparietal hypometabolism patterns on FDG-PET more strongly predicted limb or speech apraxia and VSGP. Postural instability, a defining feature of PSP, was not predicted strongly by either modality. NMF identifies important patterns such that the weighted sum approximates the original scan, in effect capturing important inherent variance in the imaging across patients. The fact that loads on these patterns did not predict a key feature strongly could have a few explanations. Postural instability may have several distinct neural mechanisms and clinical scales may be limited in their measurement of postural instability.

The presence of a PSP clinical feature was predicted more strongly than its severity. This may be because patterns of brain atrophy or hypometabolism represents disease state more so than the disease stage in PSP. A similar method was used to describe brain metabolism patterns in Alzheimer’s disease where it did predict severity in addition to subtype.^55^ Another possible reason is that available clinical scales may not be capturing severity in a way that is amenable to linear models, at least not within all individual symptom categories. Many of the MDS-UPDRS III and PSP rating scale subscores are ordinal, and the total scores result from summing over heterogenous categories. Contrast, for example, the MoCA with the PSP rating scale—the lower scores on the former likely reflects progressive cortical degeneration, whereas higher scores on the latter could result from worsening cognition, oculomotor impairment, gait and midline impairment or autonomic dysfunction, each with its own anatomical substrate.

We discovered that distinct patterns of atrophy versus hypometabolism correlated with the same clinical feature, pointing to distinct neural substrates. For example, parkinsonism (primarily bradykinesia) corresponded with subcortical basal ganglia atrophy on MRI and subcortical, precentral and superior frontal cortical hypometabolism on FDG-PET. This illustrates the complex mediation of bradykinesia through cortical and subcortical motor mechanisms.^56^ Similarly, VSGP was predicted more strongly by midbrain atrophy on MRI and frontal predominant hypometabolism on FDG-PET. These cortical–subcortical findings do concur with prior studies assessing specific symptoms. Computerized ocular motor analysis has been used to correlate saccadic and down gaze dysfunction in PSP with superior colliculus, frontal eye field and anterior cingulate hypometabolism.^31,57^ Other studies assessing gait and balance dysfunction often using instrumented gait analysis have correlated these to prefrontal–basal ganglia–midbrain circuits, with the motor thalamus mediating asymmetry.^32,58,59^ Subcortical structures, like the basal ganglia, are part of various different frontostriatal circuits and therefore featured in different imaging components.^60^ Future work using region-of-interest-based approaches may benefit from exploring more fine-grained parcellations of subcortical structures as opposed to considering them a single region.

The scale or tool used to measure a clinical deficit in PSP may influence findings in clinical neuroimaging studies. In our cohort, VSGP was better predicted by PSIS as compared to the MDS-PSP criteria alone. It is important to think about the clinical measurement tools used when assessing generalizability of results. For example, clinical scales may differ from computerized gait, balance and ocular motor analysis in terms of the depth and detail of clinical heterogeneity that may be captured. This raises an important consideration for future clinical neuroimaging investigations as digital markers are increasingly being used to categorize clinical heterogeneity in PSP.^57,61,62^

A strength of our approach was the use of a completely independent test set, which revealed that some models did not generalize. Important factors impacting generalizability include clinical heterogeneity in the sample, proportion of variant PSP phenotypes, disease stage and clinical assessment tools. Our test set was comparable in terms of disease duration and proportion of PSP Richardson syndrome, cortical and subcortical variants. As such, the results likely reflect overfitting of our prediction models in the training set, denoted by poor generalization to the test set. We took several steps to combat overfitting, including using cross-validation to select the rank for the NMF models, a mixture of L1 and L2 regularization in our regression models, and using cross-validation to select the regularization parameter. The fact that overfitting occurred nevertheless is consistent with recent literature that these regularization methods do not always work and may fail in the setting of small sample sizes where they are most needed.^63,64^ Ultimately, a much larger validation cohort with representation of all PSP variants will be required for a more robust assessment of our approach.

These important limitations should be considered when interpreting the results and to inform future studies. The fact that most of our patients had parkinsonism, dysarthria and postural instability and relatively few had speech apraxia, freezing and dystonia limited our ability to train and test models predicting these. This is a challenge faced by many groups studying PSP as the absence of validated biomarkers makes early diagnosis very challenging. By the time patients meet clinical criteria, especially those with rarer phenotypes a certain homogeneity in the core symptoms is likely if not guaranteed. This further highlights the importance of a validation/test set for evaluating clinical neuroimaging models. For example, comparing the training versus test set, the accuracy of predicting common symptoms such as dysarthria and parkinsonism declined to a lesser degree than postural instability, while the accuracy of predicting rarer symptoms declined to a lesser degree for apraxia of speech than dystonia.

We used the latest MDS-PSP diagnostic criteria, and included variant syndromes of PSP.^9^ This leads to clinical heterogeneity and limited specificity due to overlap with other neuropathologies, especially in the case of variant syndromes.^13,17,65^ Some phenotypes are indeed more associated with corticobasal ganglionic degeneration pathology.^66^ Some have argued that these criteria may be better viewed as suggestive of an underlying 4R tauopathy broadly, rather than PSP specifically.^17^ If this is true, it may explain why predicting clinical measures based on markers of neurodegeneration is difficult—it is likely that the different 4R tauopathies have heterogenous patterns of degeneration despite sometimes showing overlapping clinical manifestations. Future work using autopsy confirmed cohorts could address this.

## Conclusion

In summary, our work highlights the complexity of phenotypic diversity seen in PSP, cortical and subcortical foci of involvement and their relationship to individual symptom domains. Future research would benefit from biomarkers for early diagnosis to investigate distinct neurobiological basis of symptoms in variant PSP phenotypes. Neuroimaging modality and the tool/clinical scale used to measure clinical impairment may affect findings. Data-driven approaches offer an appropriate method to assess the PSP spectrum and the neuroanatomical substrates of its diverse presentations.

## Supplementary Material

fcae233_Supplementary_Data

## Data Availability

The data that support the findings of this study are available from the corresponding author, upon reasonable request.
