# Supplementary material for: Patterns of brain volume and metabolism predict clinical features in the progressive supranuclear palsy spectrum
Source: Brain Commun. 2024 Jul 16;6(4):fcae233. doi: 10.1093/braincomms/fcae233 (PMC11272075; doi:10.1093/braincomms/fcae233)

Supplementary Data

Table of Contents

NMF Rank Selection 2

Assessment of scanner effect on MRI: 3

Detailed description of individual MRI Components 4

Detailed description of FDG Components 9

NMF Rank Selection

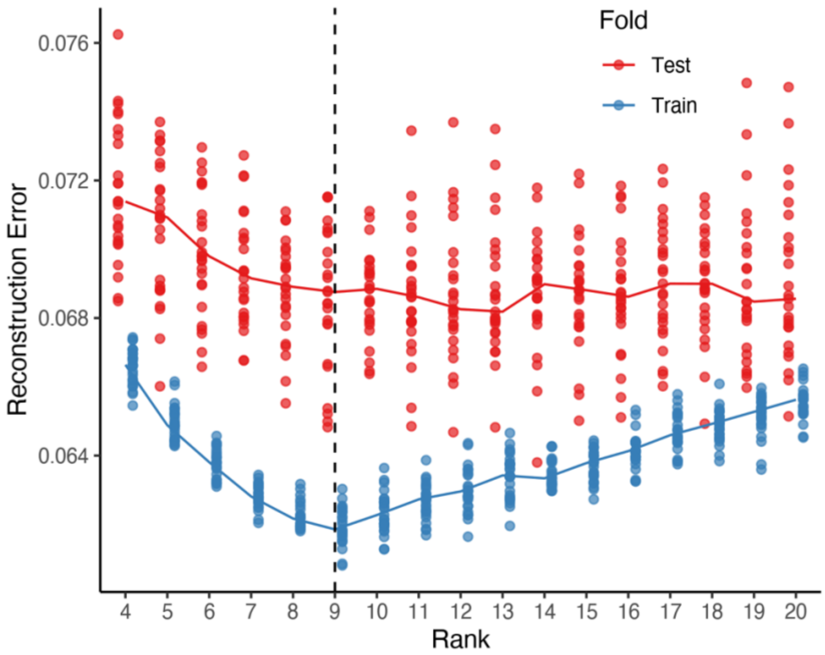

Supplementary Figure 1: Selection of MRI NMF rank: The median reconstruction loss was minimized at a rank of 9

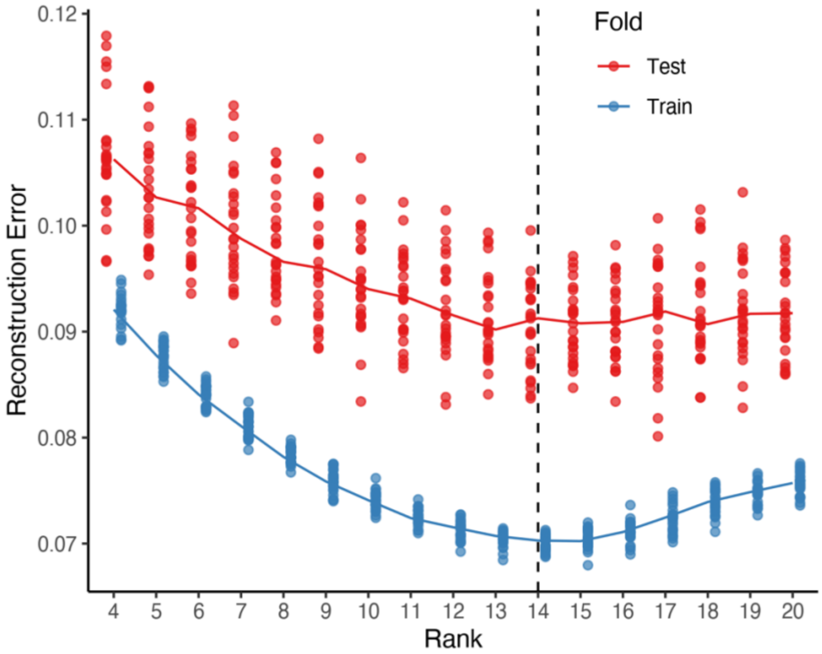

Supplementary Figure 2 : Selection of FDG NMF rank: The median reconstruction loss was minimized at a rank of 14

Assessment of scanner effect on MRI:

In the training set 54 patients had 3T MRI on GE scanners, and rest on Siemens. In the test set 4 had MRI on GE scanners and 16 on Siemens. All test and training FDG-PET scans were done on a GE PET/CT scanner. There were 2 components that exhibited a scanner effect, both in the direction of larger loads for participants scanned on Siemens.

- Component 1 (p-adjusted = 0.000179 for t-test comparing GE vs Siemens)
- Component 4 (p-adjusted = 0.0134 for t-test comparing GE vs Siemens)
-

However, such a difference does not necessarily imply a bias in the results – that requires that there be differences on clinical measures in those scanned on GE vs Siemens.
For binary measures, the following measures had the most evidence for a different between groups:

Supplementary Table 1

	Proportion (GE)	Proportion (Siemens)	p	p-adjusted (FDR)
FTD-syndrome (MDS)	0.143	0.351	0.04	0.3
Limb Apraxia Present (PSPRS)	0.442	0.216	0.06	0.3

Neither component was part of the model for FTD-syndrome. For Limb Apraxia, component 1 was selected, but with a coefficient that was very low – 0.048 of the max coefficients in that model (i.e., contributing a lot less than other components).
For continuous MRI measures, the following measures had the most evidence for a different between groups:

Supplementary Table 2

	Median (GE)	Median (Siemens)	p	p-adjusted (FDR)
Eyes Horizontal (PSPRS)	2	1	0.001	0.04
Eyes Total (PSPRS)	9	6	0.003	0.05
PSPRS Total Score	47	37	0.006	0.06

Component 4 was not selected for any of the three models (coefficient = 0) whereas Component 1 formed part of all three. However, the R-square for models predicting these measures were among the worst (Eyes) or average (PSPRS Total) in the training set.

This argues strongly against results being influenced by scanner effect. We do not think it is good practice to hand pick components as it goes against the aims of a data driven approach, where high dimensional data are decomposed and then regularized models are used to select relevant components.

Detailed description of individual MRI Components

Deeper reds indicated more positive weights at that voxel, with weights representing the probability of tissue being present at that voxel. For example, if a patient had high loads on component 1, they would be expected to have higher tissue probability in the red areas, and conversely lower loads would indicate low probability of tissue there, i.e., atrophy.

Component 1

Bilateral temporal, with lower weights in biparietal areas

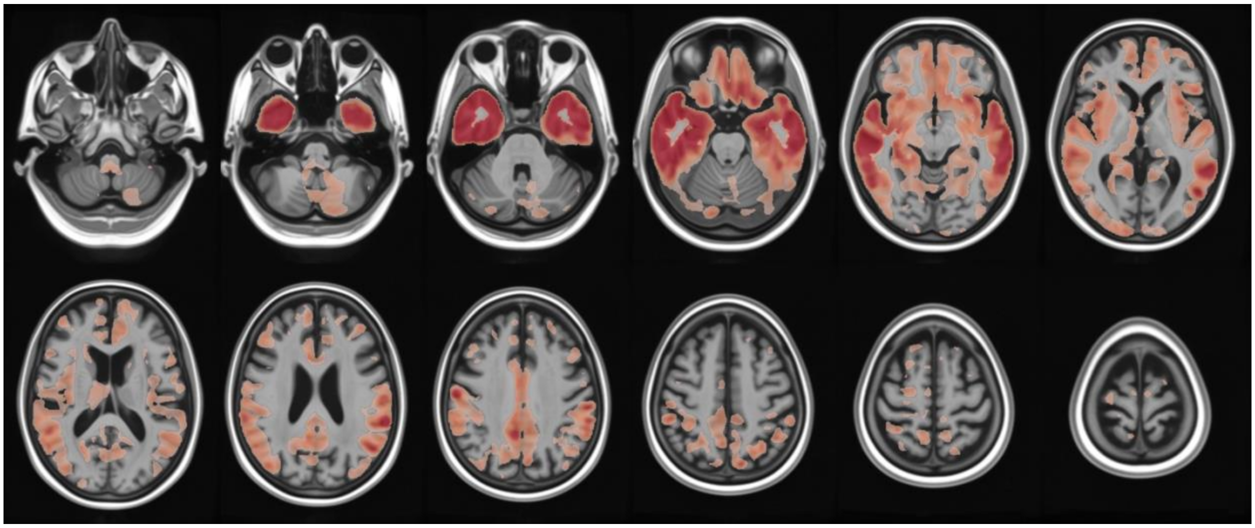

Component 2

Bilateral occipital and medial occipitotemporal and occipito-parietal

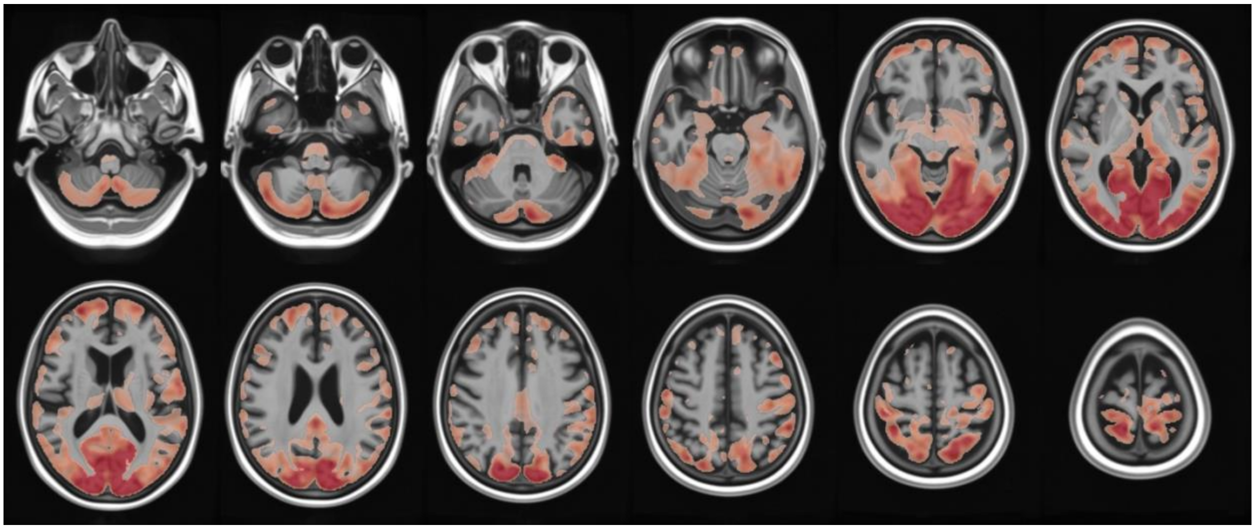

Component 3

Widespread deep cortical and juxtacortical weights

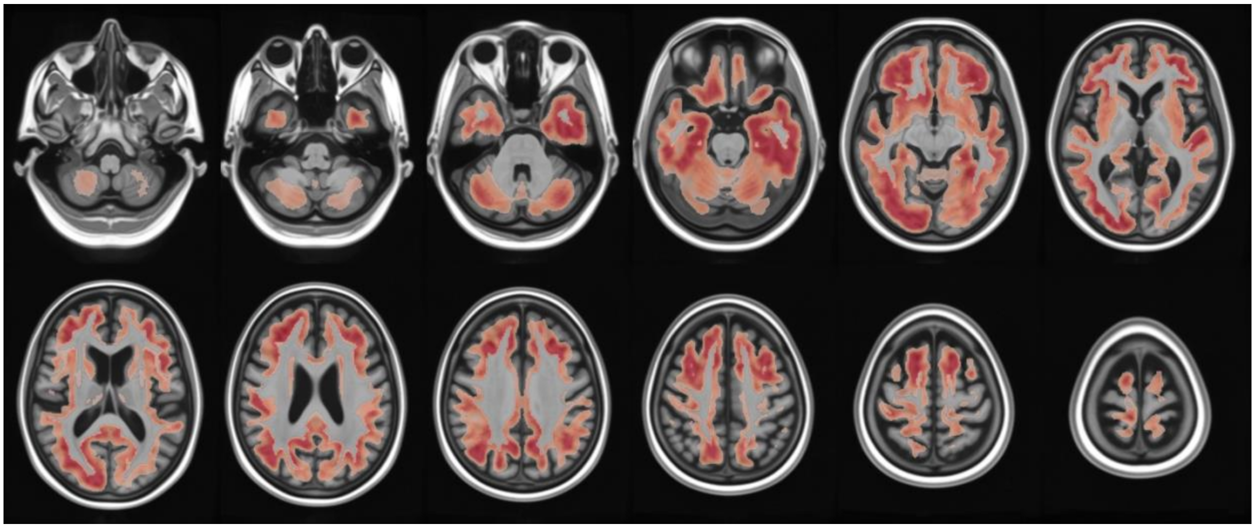

Component 4

Cerebellum and brainstem, with additional subcortical gray matter and deep cortical/juxtacortical weights

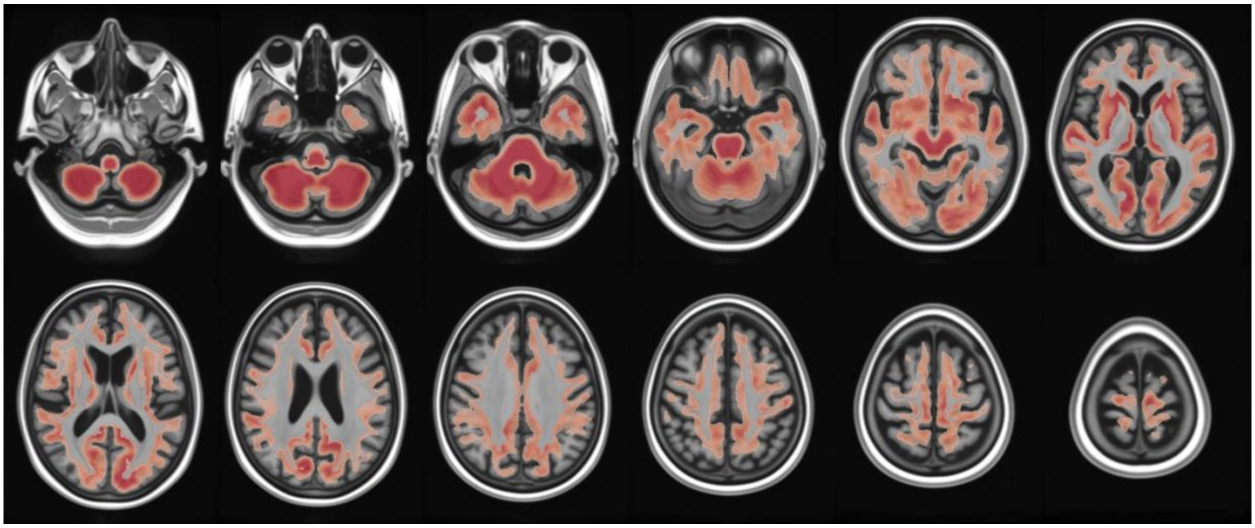

Component 5

Brainstem and subcortical gray matter, with additional right greater than left frontoparietal weights

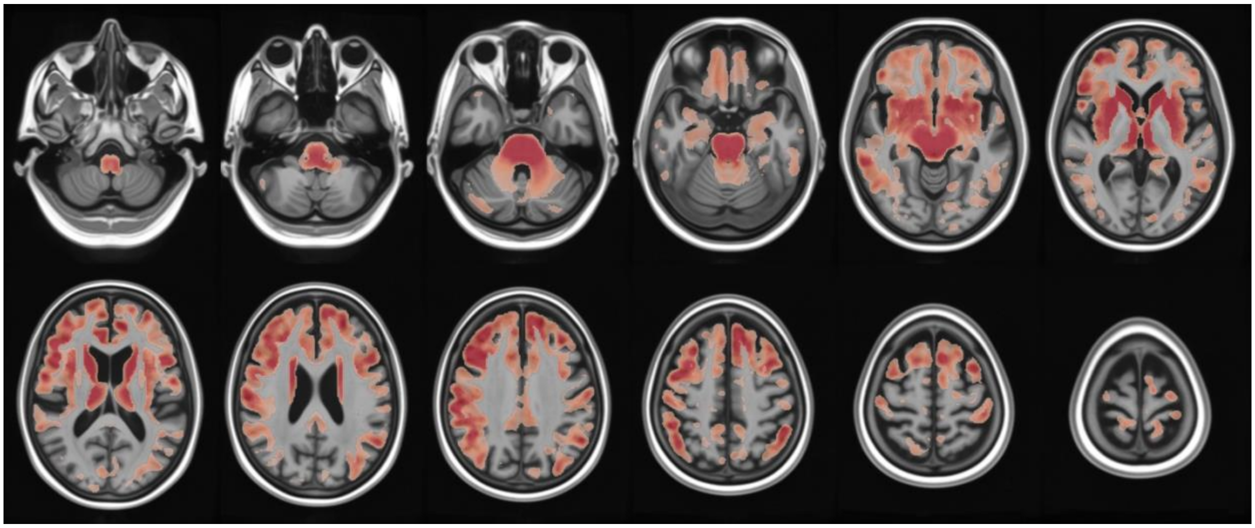

Component 6

Bilateral posterior-lateral temporal, and biparietal weights.

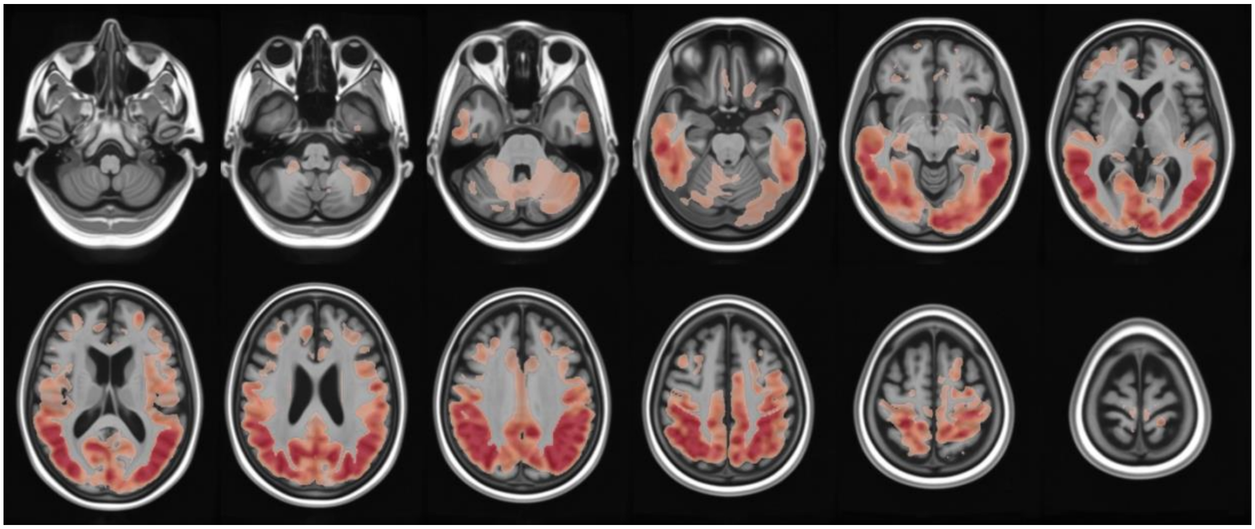

Component 7

Bifrontal weights

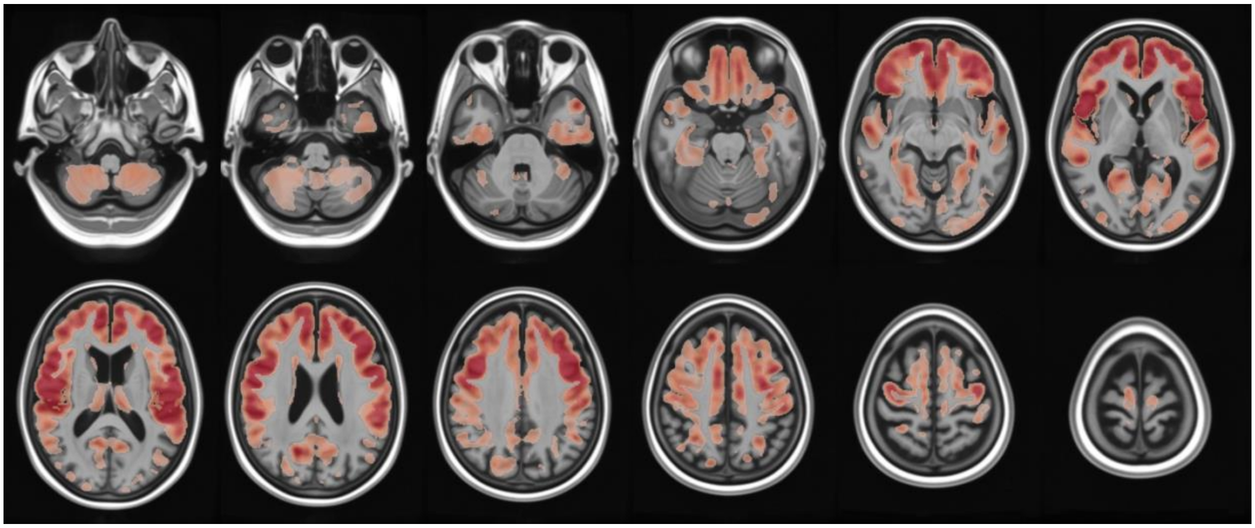

Component 8

Cerebellum

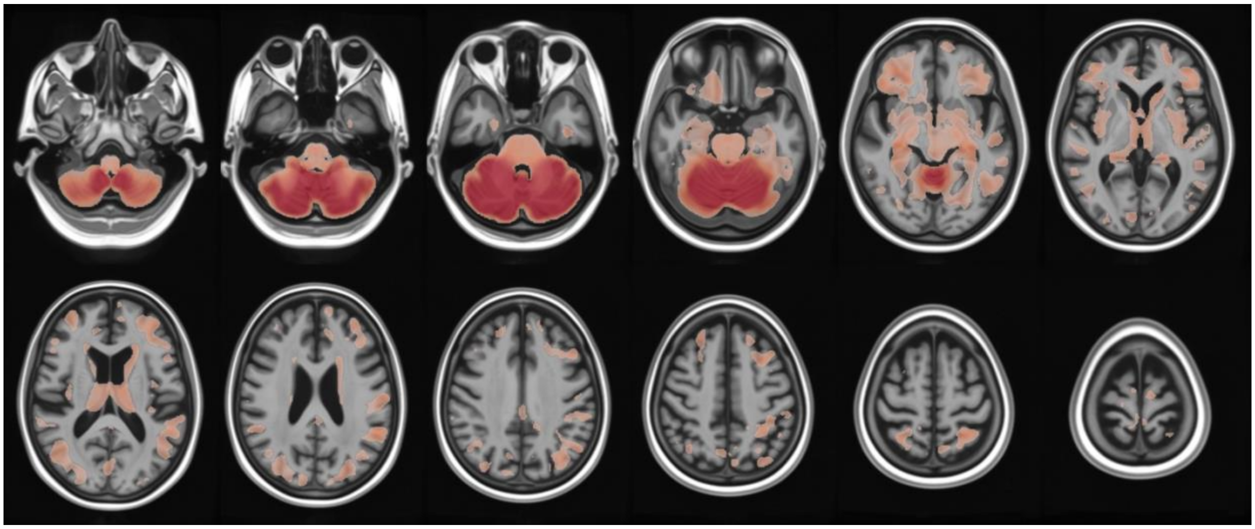

Component 9

Bilateral superior cortical regions, including lateral precentral gyrus

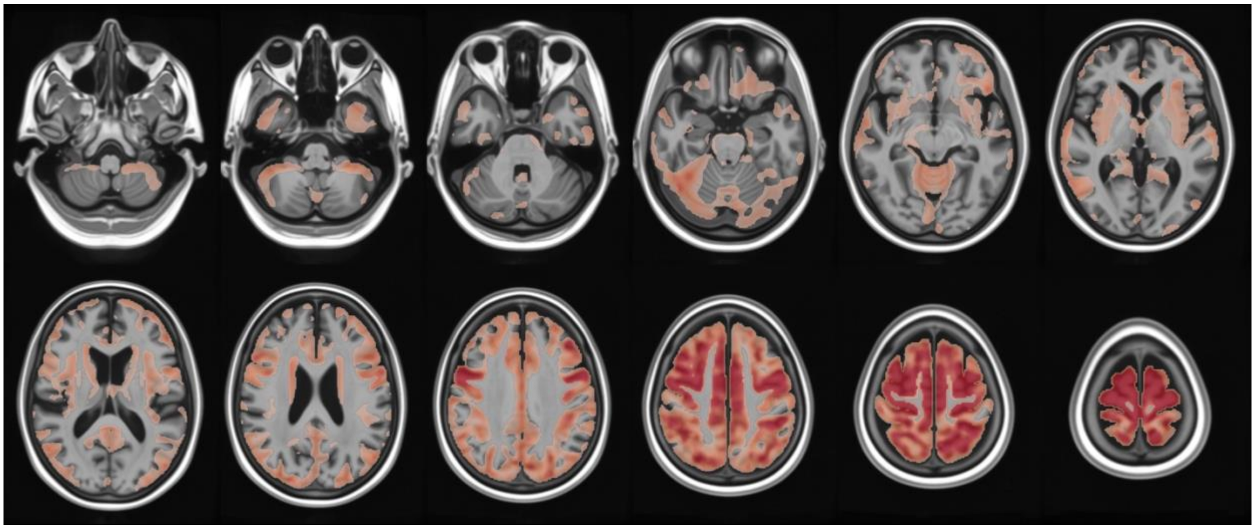

Detailed description of FDG Components

Deeper reds indicated more positive weights at that voxel, with weights representing the FDG uptake (SUVR) at that voxel. For example, if a patient had high loads on component 1, they would be expected to have higher SUVR values in the red areas, and conversely lower loads would indicate lower SUVR there, i.e., hypometabolism.

Component 1

Biparietal predominant, with additional weights across lateral frontal and temporal regions

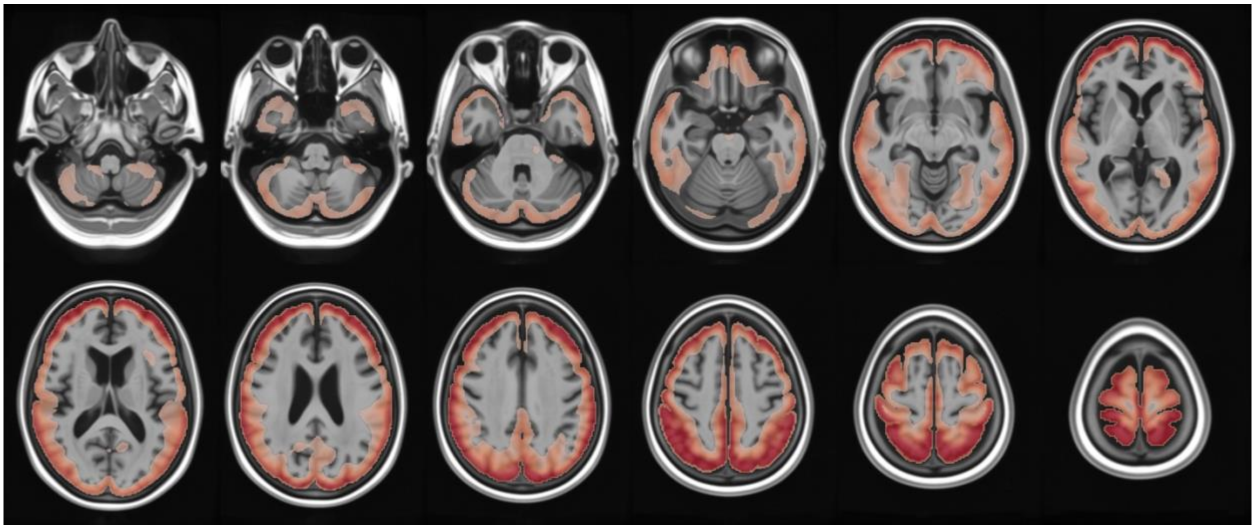

Component 2

Right greater than left lateral frontal, parietal, and temporal weights

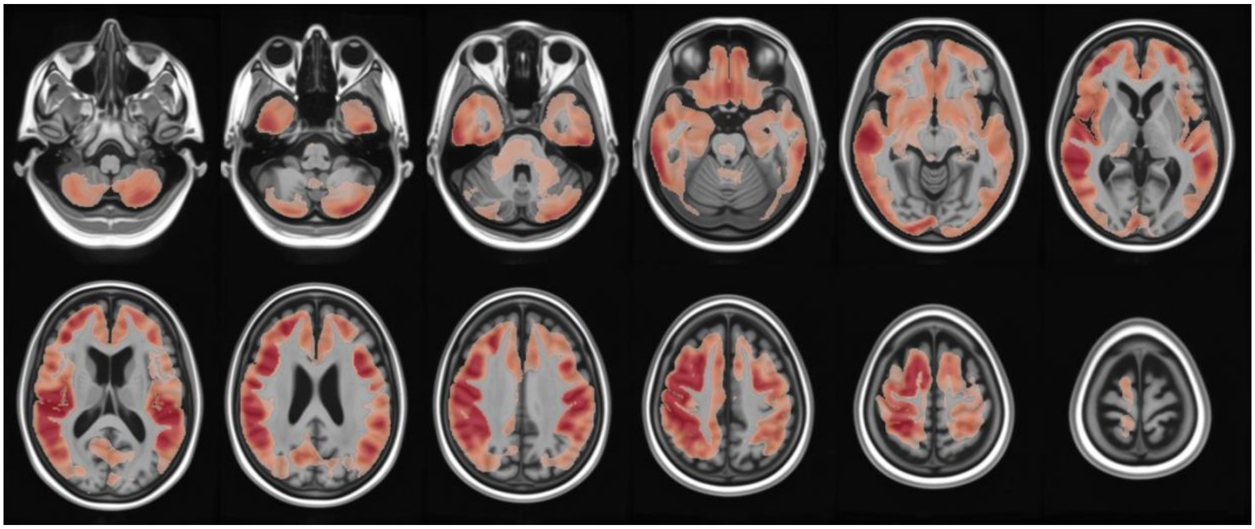

Component 3

Bilateral parietal-frontal, right more than left

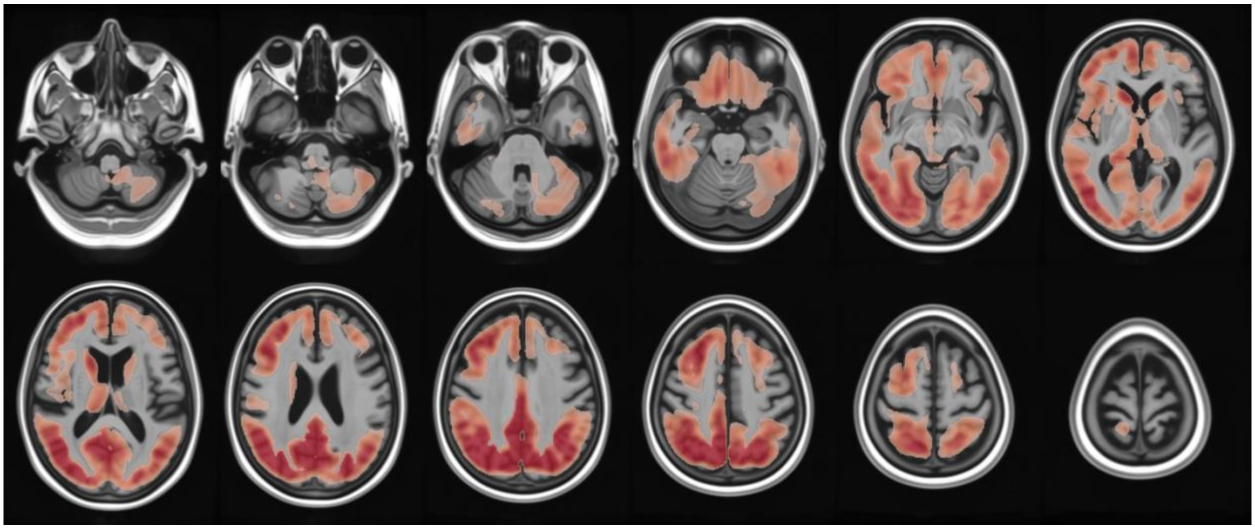

Component 4

Hight parietal and frontal, as well as subcortical and to a lesser extent lateral parietal weights

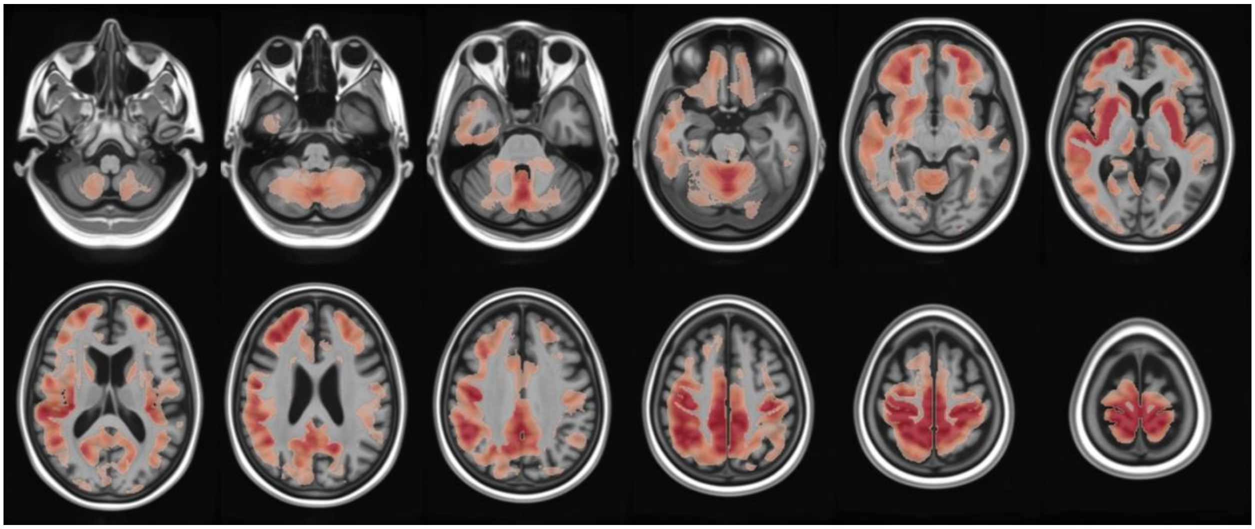

Component 5

Bitemporal as well as subcortical gray matter and deep cortical/juxtacortical weights.

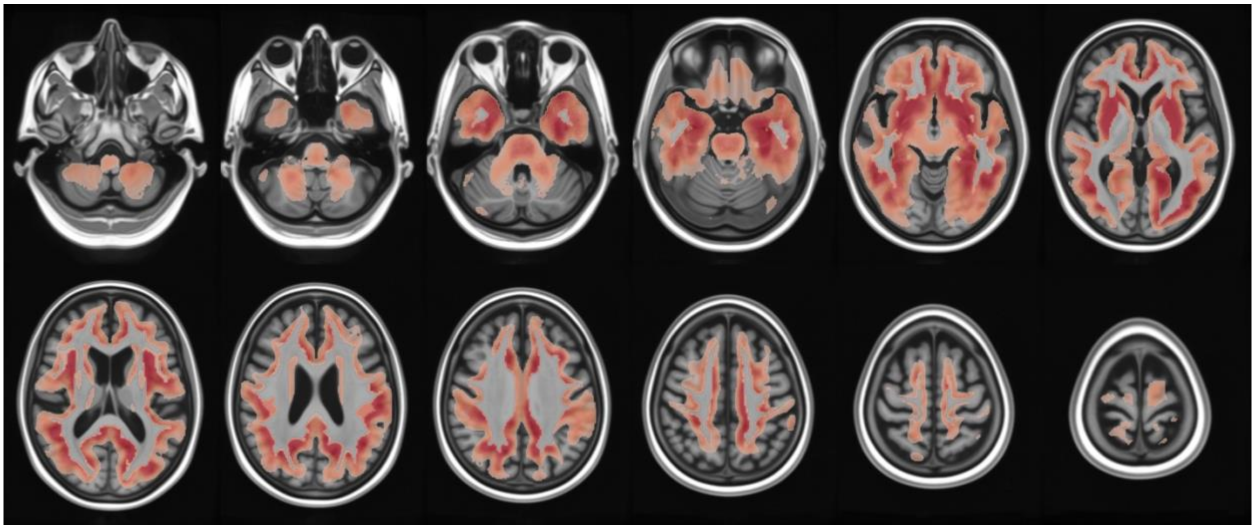

Component 6

Bilateral subcortical gray, insulae, medial occipital-parietal predominant weights

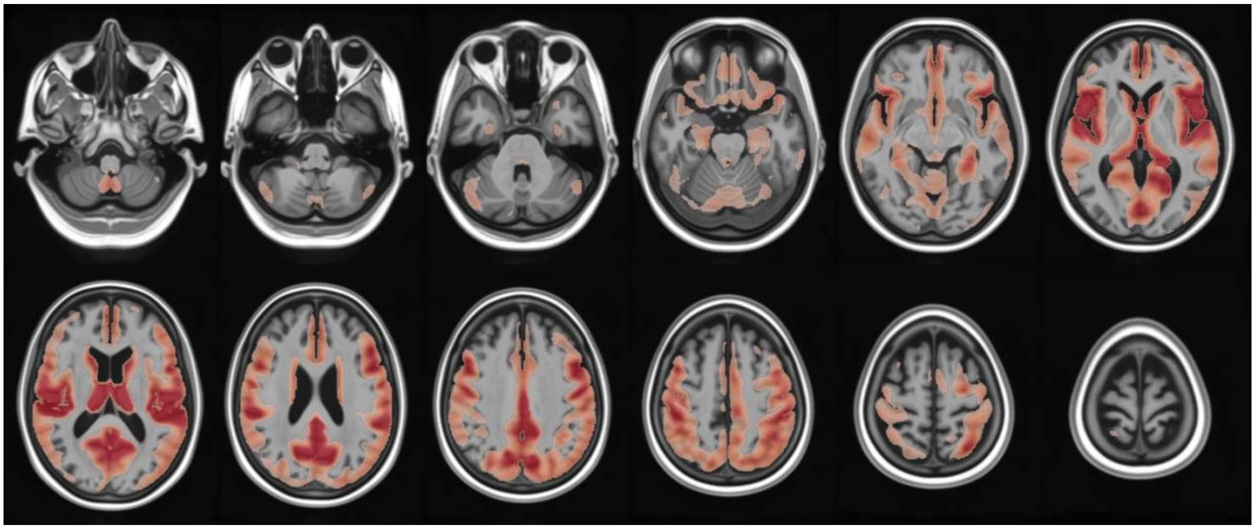

Component 7

Cerebellum with brainstem and subcortical gray

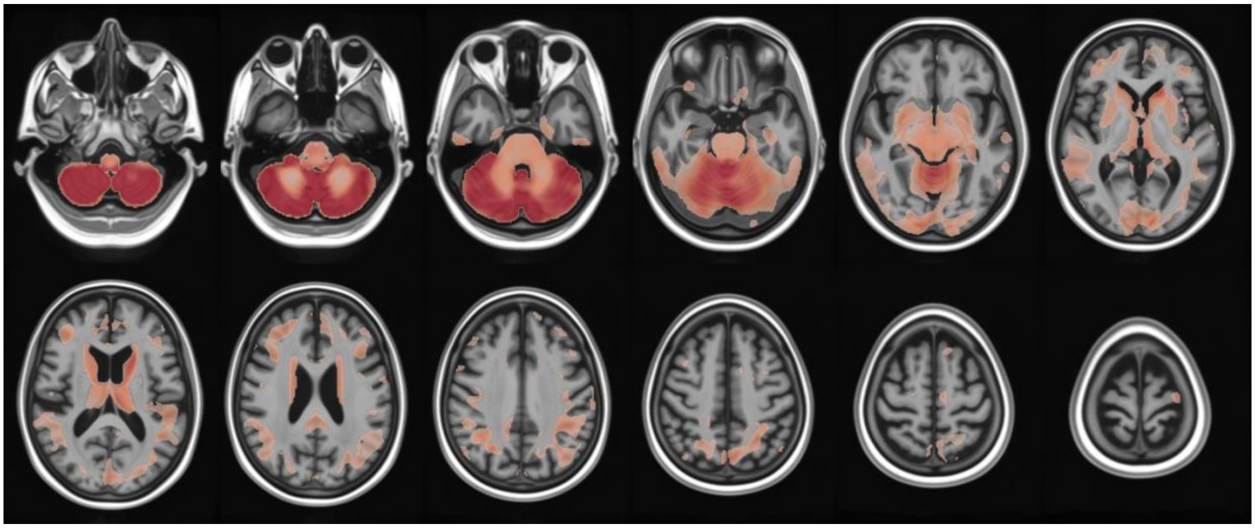

Component 8

Bilateral orbitofrontal and occipitotemporal, posterior cingulate, precuneus, and lateral parietal.

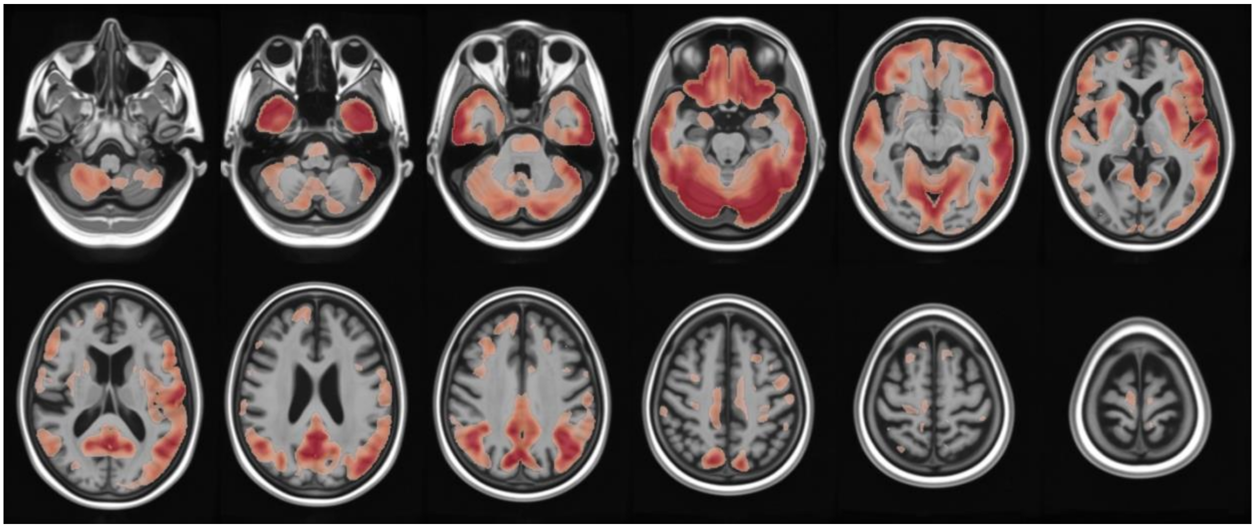

Component 9

Left greater than right parietal-frontal, and to a lesser extent occipital and lateral temporal

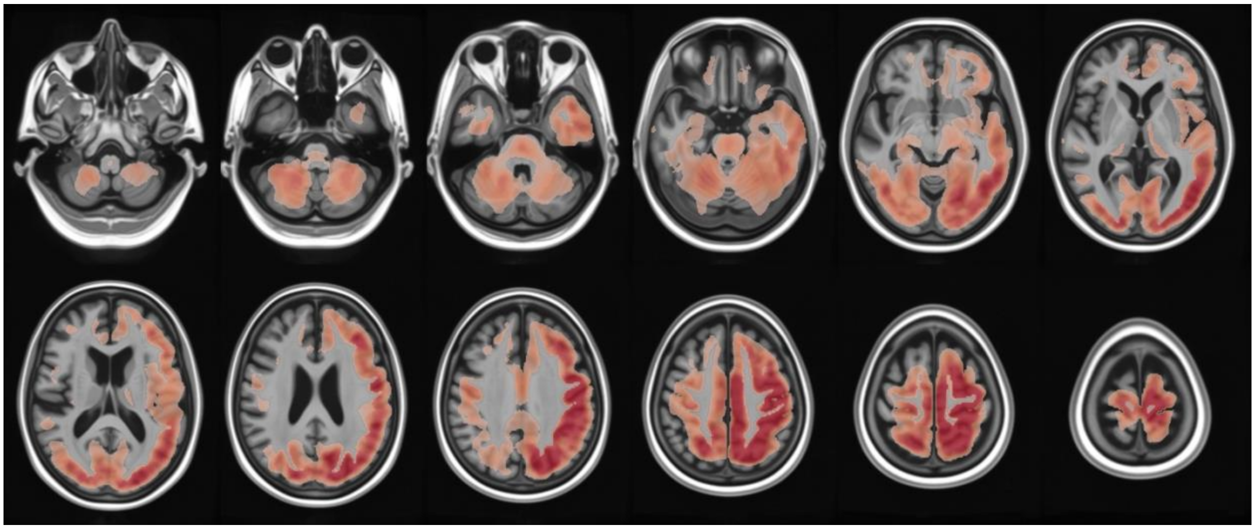

Component 10

Bilateral medial and inferior occipital

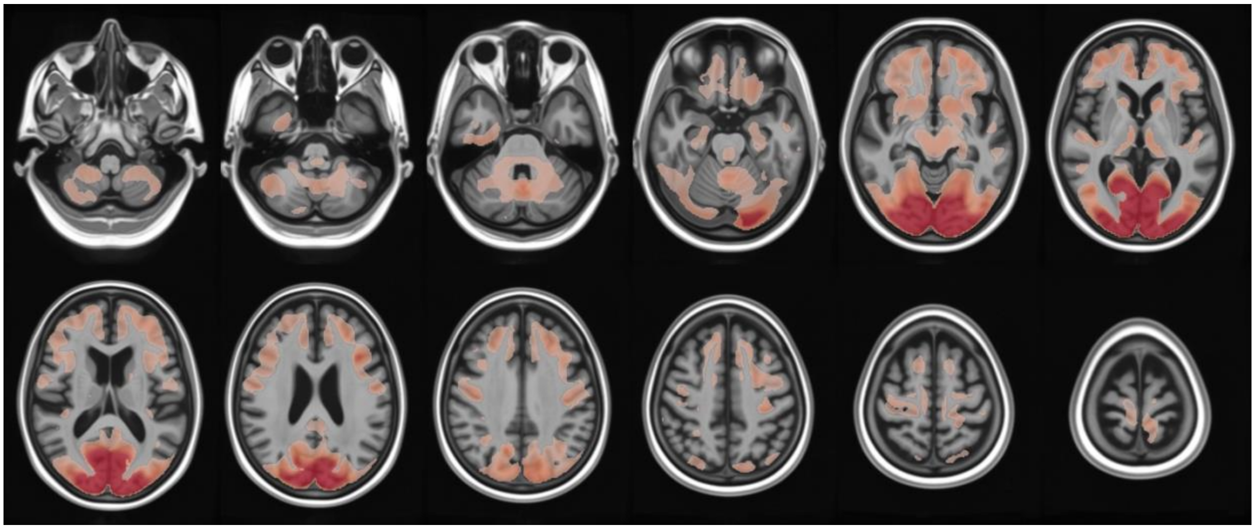

Component 11

Cerebellum with some occipital and frontal-temporal predominantly on the right

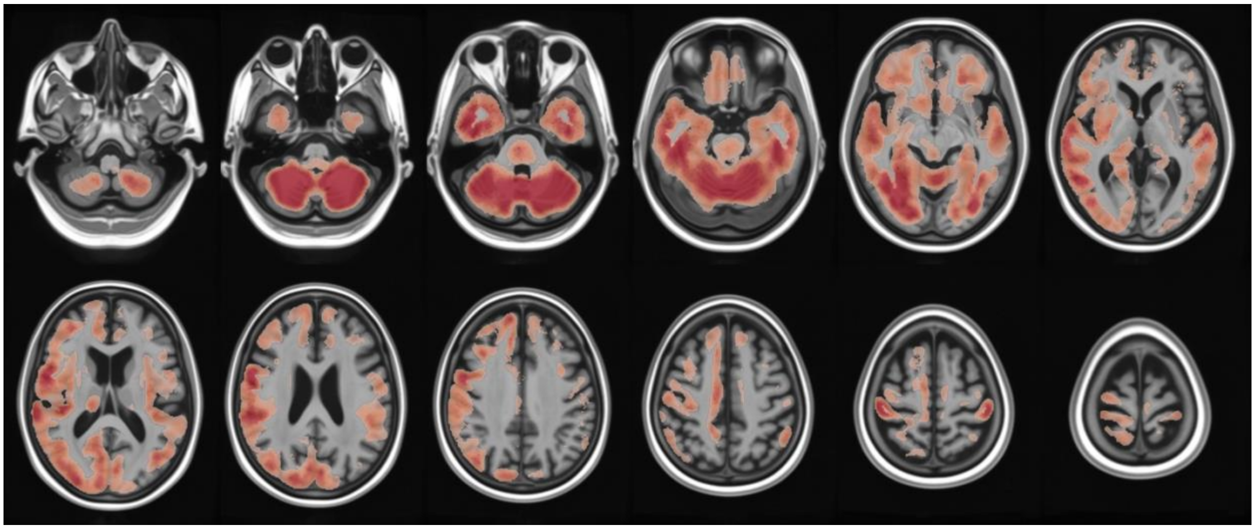

Component 12

Bifrontal with some superior cerebellum

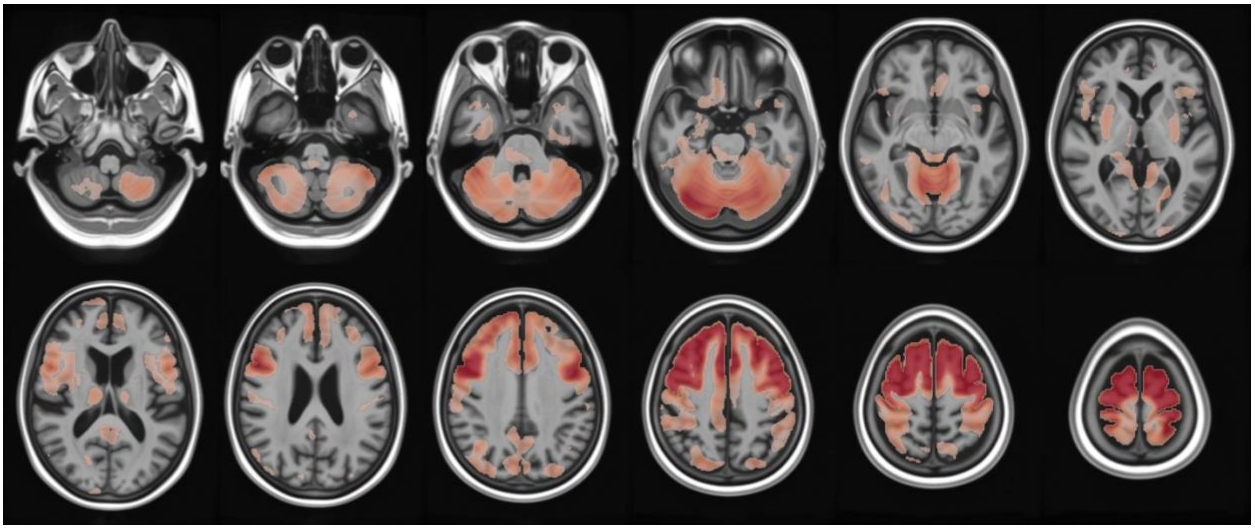

Component 13

Bilateral medial frontal and occipital, as well as orbitofrontal, subcortical, and insular.

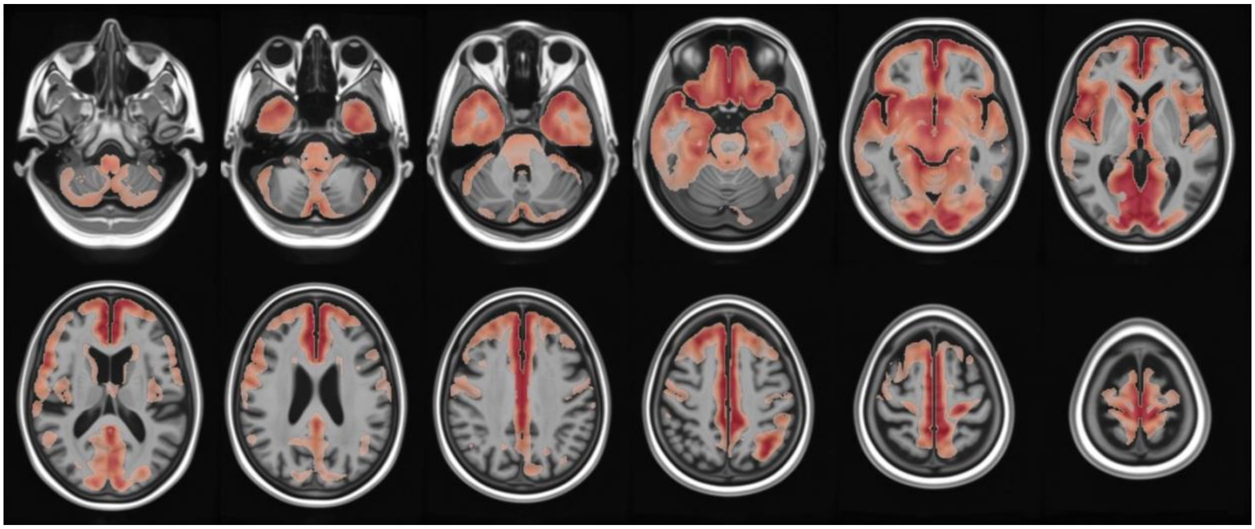

Component 14

Bilateral lateral frontal, subcortical gray, and midbrain, more so on the left

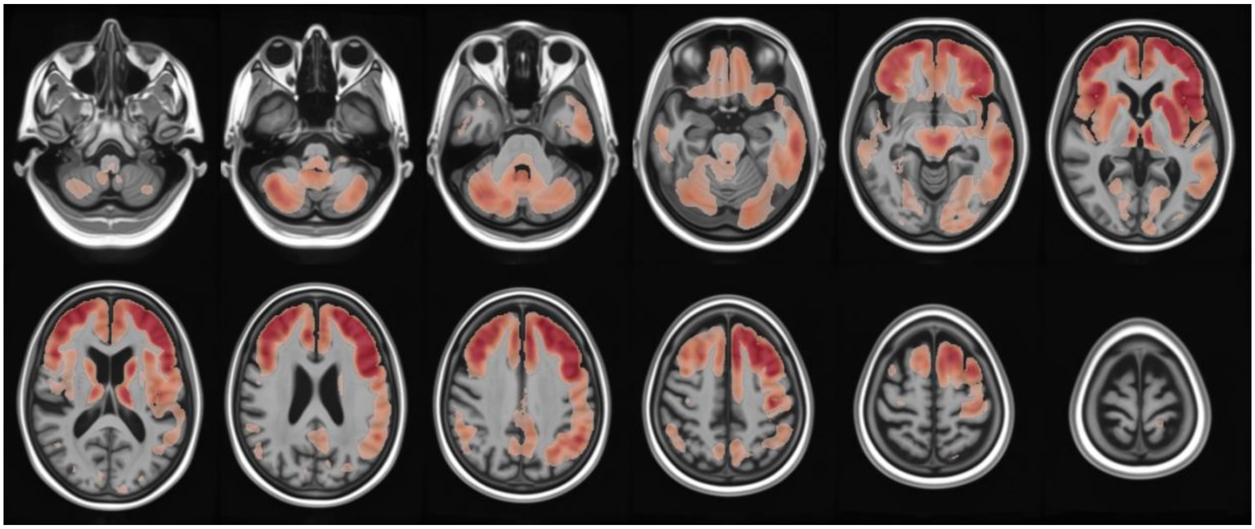

Supplement: fcae233_Supplementary_Data [file fcae233_supplementary_data.pdf]
